# Associations of Maternal Polyunsaturated Fatty Acids With Telomere Length in the Cord Blood and Placenta in Chinese Population

**DOI:** 10.3389/fnut.2021.779306

**Published:** 2022-01-28

**Authors:** Xuanyi Liu, Qiaoyu Shi, Xiuqin Fan, Hang Chen, Na Chen, Yurong Zhao, Kemin Qi

**Affiliations:** ^1^Key Laboratory of Major Diseases in Children, Laboratory of Nutrition and Development, Ministry of Education, Beijing Pediatric Research Institute, Beijing Children's Hospital, Capital Medical University, National Center for Children's Health, Beijing, China; ^2^Department of Obstetrics and Gynecology, Fuxing Hospital, Capital Medical University, Beijing, China

**Keywords:** maternal n-3 fatty acids, newborns, telomere length, telomerase reverse transcriptase, DNA methylation

## Abstract

Few studies have investigated the correlation between maternal polyunsaturated fatty acids (PUFAs) and telomeres in offspring, and the underlying influential mechanisms. In this study, we assessed the associations of maternal PUFAs with telomere length (TL) and DNA methylation of the telomerase reverse transcriptase (TERT) promoter in the cord blood and the placenta. A total of 274 pregnant women and their newborn babies were enrolled in this study. Maternal blood before delivery, the cord blood, and the placenta at birth were collected. Fatty acids in maternal erythrocytes and cord blood cells were measured by gas chromatography (GC). TL in the cord blood and the placenta was determined using real-time quantitative PCR (qPCR) by calculating the product ratio of telomeric DNA to the single-copy gene β-globin. The TERT promoter methylation was analyzed by DNA bisulfite sequencing. The associations of maternal fatty acids with TL were analyzed by univariate and multivariate regression. We found that low concentrations of docosapentaenoci acid (DPA, C22: 5n-3) and total n-3 PUFAs, adrenic acid (ADA, C22: 4n-6), and osbond acid (OA, C22: 5n-6) and high concentrations of linoleic acid (LA, C18: 2n-6) in maternal erythrocytes were associated with the shortened TL in cord blood cells (estimated difference in univariate analysis −0.36 to −0.46 for extreme quintile compared with middle quintile), and that low concentrations of cord blood docosahexaenoic acid (DHA, C22: 6n-3) were related to the shortened TL in cord blood cells. Differently, high concentrations of α-linolenic acid (LNA, C18: 3n-3), eicosatrienoic acid (EA, C20: 3n-3), DHA, and γ-linoleic acid (GLA, C18:3n-6) in maternal erythrocytes were associated with the shortened TL in the placenta (estimated difference in univariate analysis −0.36 to −0.45 for higher quintiles compared with the middle quintile). Further examination demonstrated that the concentrations of DHA and total n-3 PUFAs in maternal erythrocytes had positive associations with DNA methylation of the TERT promoter in the cord blood instead of the placenta. These data suggest that maternal PUFAs are closely correlated to infant TL and the TERT promoter methylation, which are differently affected by maternal n-3 PUFAs between the cord blood and the placenta. Therefore, keeping higher levels of maternal n-3 PUFAs during pregnancy may help to maintain TL in the offspring, which is beneficial to long-term health.

## Introduction

The developmental origins of health and disease (DoHaD) hypothesis posit that nutritional factors and environmental stimuli in the early life stage have important long-term consequences for subsequent health and susceptibility of chronic diseases, in particular, diabetes, obesity, and cardiovascular disease (1, 2). Two developmental pathways explain the DoHaD concept—the adaptive programming resulting from a mismatch between *in utero* and postnatal environments, and the direct exposure of the fetus to environmental factors, causing increased disease risk later in life (3, 4). It has been addressed that the effects of these non-genetic factors on offspring' health decades later are primarily mediated through epigenetic modification on gene expression, including DNA methylation, histone modification, non-coding RNA, and genomic imprinting (5, 6). Meanwhile, under suboptimal intrauterine conditions, telomere system can be altered by the programming actions of stress-related maternal–placental–fetal oxidative, immune, endocrine, and metabolic pathways, and may ultimately accelerate cellular dysfunction, aging, and disease susceptibility over the lifespan (7).

Telomeres, the specialized DNA-protein structures located at the end of eukaryotic chromosomes, are composed of non-coding double-stranded repeats of guanine-rich tandem DNA sequences and shelterin protein structures (8), which function to maintain chromosome stability and prevent the loss of genomic information caused by the semiconservative replication of DNA during cell division (9). Telomere length (TL) is maintained by the active telomerase consisting of a single long non-coding telomerase RNA (TER), telomerase reverse transcriptase (TERT), and other proteins, which prevent telomere shortening during DNA duplication by adding telomeric repeat DNA to chromosome ends (10). It has been found that telomerase activity, being higher in proliferative cells (germline cells, stem cells, activated lymphocytes, hematopoietic cells, immortal cells, and certain types of cancer cell populations), varies with the cell-cycle stage and other factors. However, in most somatic cells after birth telomerase activity is mostly undetectable (7, 11, 12). Strong pieces of evidence have indicated that TL and telomerase activity are associated with a host of genetic and non-genetic factors, including age, sex, socio-economic status, race/ethnicity, body mass index (BMI), infection, diet/nutrition, physical activity, sleep, stress, social relationships, and diseases (13). The consumption of fruits and vegetables, Mediterranean diet, and antioxidant nutrients is mainly associated with a longer TL (14). The underlying mechanisms for altered TL are thought to be involved in several pathways, including rearrangement and/or promoter mutations, epigenetic modification on TERT, and inflammation and oxidative damages, but still remain to be investigated (15–17).

Telomere length is strictly regulated in the placenta and embryo by telomerase, playing a crucial role in fetal development (18). It has been reported that TL and telomerase activity in embryos are related to the common genomic stability at the cleavage stage of human development (19). More importantly, TL in early life rather than in adulthood might be more important in predicting lifespan because it has more effects on subsequent tissue function (20). Several studies have demonstrated inverse associations between blood TL in newborns and children and maternal exposure to environmental toxicants (air pollution, toxic metals, and secondhand smoke) during pregnancy (21–23). Telomeres may also be susceptible to poor or unbalanced nutrition. It has been reported that TL in the offspring is positively related to folate, vitamin D, and caffeine concentrations in maternal diet (24), but negatively related to maternal saturated fat intake during pregnancy and prepregnancy BMI (25, 26). In addition, n-3 polyunsaturated fatty acids (n-3 PUFAs) may be a factor that prevents telomere shortening in cell division, for which they have anti-inflammatory and anti-oxidative effects, particularly, docosahexaenoic acid (DHA) and eicosapentaenoic acid (EPA) (27–29). However, few studies have investigated the correlation between maternal n-3 PUFAs and telomeres in newborns and after birth. In the Seychelles Child Development Study, PUFA status and methylmercury exposure were not associated with TL in mothers and their children (30). A double-blind randomized control trial found that prenatal n-3 PUFA supplementation did not affect offspring TL (31). The quantity of dietary n-3 PUFA intake both in the general population and pregnant women varies with different regions and countries, which may affect the results from different studies (32). Meanwhile, the placenta and the cord blood have different lifespans and functions and may differ in sensitivity to maternal nutrition. In comparison with the placenta cells that undergo cellular senescence during pregnancy induced by oxidative stress, inflammatory response, etc., cord blood cells are primarily at early hematopoietic stem cells with a high differentiation potential such that they can better maintain TL (17, 33, 34). Therefore, this study aimed to ascertain the association of maternal PUFAs with TL and TERT DNA methylation in the cord blood cells and the placenta in Chinese women.

## Subjects and Methods

### Subjects

This study was based on a mother–child cohort in the Department of Obstetrics and Gynecology of Fuxing Hospital, Capital Medical University. Pregnant women from the Chinese Han population aged 22–35 years in the third trimester were recruited to participate in the study between July 2020 and June 2021. Women who had metabolic, endocrine, and hereditary disorders, and diseases treated with drugs were excluded. Finally, out of a total of 290 pregnant women, 274 pregnant women and their newborn babies were enrolled in the study. This study has been registered on the Clinical Trials.gov (No: ChiCTR-OCH-14004900) and approved by the Institutional Review Board and Committee on Human Research at Fuxing Hospital, Capital Medical University (2020FXHEC-KSKY005). The written informed consent from all women was obtained after oral and written explanation of this study.

### Data and Sample Collection

All pregnant women were invited to complete a general form to obtain the information (age, body weight and height, race, education, social and economic status, diseases, medicines used, etc.) from themselves and their husbands, and a semiquantitative food frequency questionnaire to obtain food intake and supplementation of nutrients, including DHA during the whole period of pregnancy. The gestational age, gender, birth weight, and the length of newborns were collected from medical records. Maternal venous blood samples were collected in EDTA anticoagulant tubes before delivery. Plasma and blood cells were fractionated by centrifugation (3,000 rpm for 15 min at room temperature), exactly at 15 min after blood withdrawal. During delivery, the amnion of the placenta was removed, and a biopsy of 2 cm × 2 cm × 2 cm was cut out nearly 5 cm from the umbilical cord insertion and 1.0–1.5 cm below the fetal membrane, so as to obtain homogenous samples from the placental villous parenchyma. Then, the residual maternal blood in the placenta was removed by washing with sterile phosphate buffer saline. The babies' umbilical vein blood was collected with EDTA as an anticoagulant in the labor room or operation theater immediately after delivery by trained midwives, and processed in the same way as the maternal blood samples. The separated cord blood cells consisted of complex varieties of cells, including stem cells, leukocytes, and erythrocytes. All samples including placenta, plasma, and blood cells were stored at −80°C until analysis.

### Fatty Acid Analysis

Fatty acids in maternal erythrocytes and cord blood cells were measured by gas chromatography (GC) based on fatty acid methyl esters (FAMEs), which were prepared according to a modified method of Lepage (35, 36). Briefly, 2 ml of a mixture solution of methanol n-hexane (4:1, vol/vol) was added to 100 μl of erythrocytes containing C15:0 (an internal standard) and 2 μl of butylated hydroxyanisole (BHT) (20 mM) to prevent lipid oxidation, and then 0.2 ml of acetyl chloride was slowly added. After heating at 100°C for 1 h, 5 ml of 6% K_2_CO_3_ solution was added to the tube, mixed on a vortex, and centrifuged, and the clear n-hexane top layer containing FAMEs was transferred to a GC autosampler vial for analysis. FAMEs were analyzed on an Agilent 6,890 N GC system, and the quantity of fatty acids was expressed as the percentage (%) (wt/wt) of total fatty acids as described in our previous studies (37, 38). Then, concentrations of n-3 PUFAs in maternal erythrocytes and cord blood cells were analyzed with quintile distributions.

### TL Determination

Genomic DNA was isolated and purified from 25 μl of cord blood nucleated cells and 25 mg of placental tissues using the TIANamp Micro DNA kit (Tiangen Biotech, Beijing, China) and an Animal Tissue DNA kit (Simgen Biotech, Hangzhou, China), respectively. TL was determined using the real-time qPCR on a CFX96 Touch TM Real-Time PCR Detection System (Bio-Rad) by measuring the ratio of telomeric DNA product to the single-copy gene product (T/S ratio), which was calculated by 2^−(Δ*Ctt*−Δ*Cts*)^/mean of all plates 2^−(Δ*Ctt*−Δ*Cts*)^ = 2^−Δ*ΔCt*^/mean 2^−Δ*ΔCt*^. The primers for the telomere PCR were tel 1: 5′-GGTTTTTGAGGGTGAGGGTGAGGGTGAGGGTGAGGGT-3′ and tel 2: 5′-TCCCGACTATCCCTATCCCTATCCCTATCCCTATCCCTA-3′, and the primers for the single-copy gene human β-globin were Hbg1: 5′-GCTTCTGACACAACTGTGTTCACTAGC-3′ and Hbg2: 5′-CACCAACTTCATCCACGTTCACC-3′ (38, 39).

### DNA Bisulfite Conversion and Sequencing

**Figure 1:**
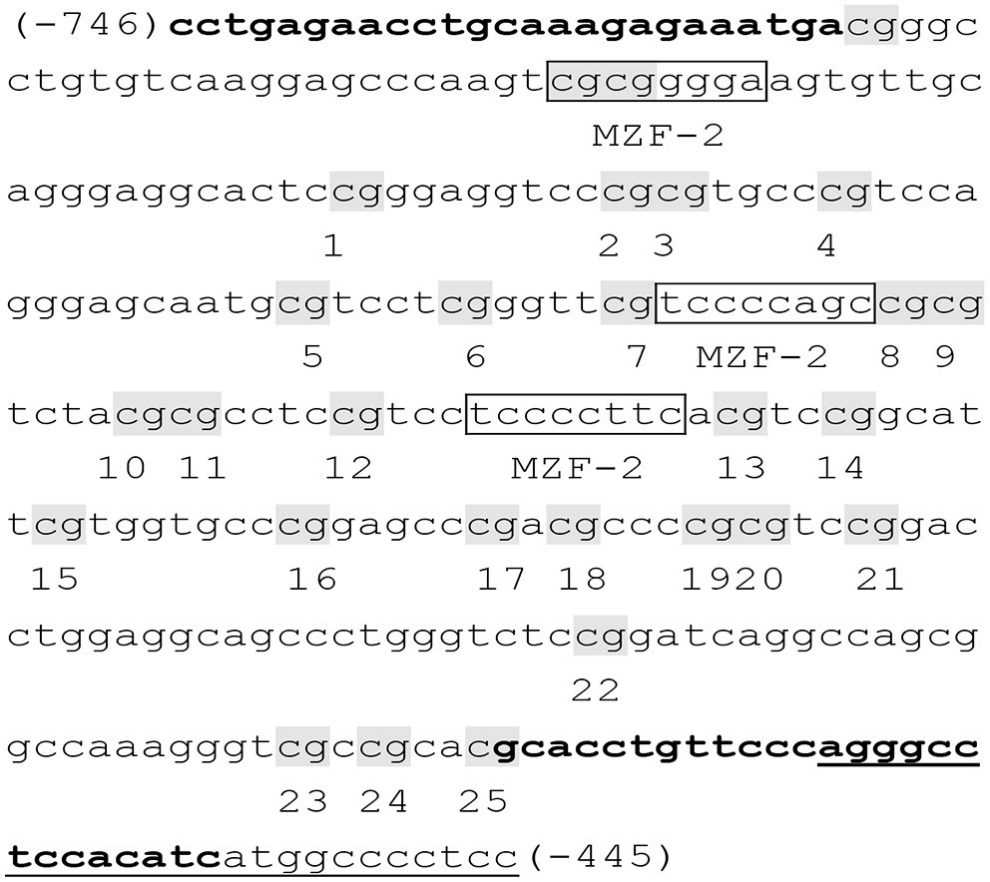
Primer sequences of the TERT promoter (38). The examined TERT promoter region includes nucleotides (nts) 1254,147–1253,148 and spans 25 CpGs within nts −746 to −445 (positions are given relative to the transcription start site), containing 3 binding sites (boxed) for myeloid-specific zinc finger protein 2 (MZF2). CpG sites are labeled with shadow and numbered from 1 to 25. Primers were shown in bold, and the primer outer R was underlined.

A total of 25 CpG sites of the TERT promoter spanning nucleotides (nts) −746 to −445 (positions are given relative to the transcription start site) within nts 1254,147–1253,148 (Figure 1) (38). The obtained sequence data (accession NC_000005) in the GenBank database (http://www.ncbi.nlm.nih.gov) have been examined with binding sites for myeloid-specific zinc finger protein 2 (MZF2). Bisulfite sequencing was used to assess DNA methylation of the TERT promoter. Genomic DNA was given bisulfite modification with the EZ DNA Methylation TM kit (ZYMO Research, USA). Then, nested PCR was conducted on the methyl-modified DNA with special primers, and the products were sequenced directly. Specific primers for the TERT promoter were as follows: F, 5′- TTTGAGAATTTGTAAAGAGAAATGA-3′; inner R, 5′-AATATAAAAACCCTAAAAACAAATAC-3′; and outer R, 5′-AAAAAAACCATAATATAAAAACCCT-3′ (38). DNA methylation was calculated from the amplitude of cytosine and thymine within each CpG dinucleotide, C/(C+T), as described by Lewin et al. (40).

### Statistical Analysis

All analyses were performed with SPSS version 20.0. The Kolmogorov–Smirnov test was used to evaluate whether the data are normally distributed. Variables conforming to normal distribution were presented as mean and SD. The characteristics for parents (maternal age, prepregnancy BMI, gestational weight gain, folic supplement, fish oil supplement, and paternal age and BMI) and newborns (gestational age, gender, birth weight and length, and BMI), outcome variables, and fatty acid concentrations were descriptively analyzed. The linear regression analysis was conducted to explore the univariate associations between individual fatty acid concentrations in maternal erythrocytes and TL in the cord blood cells and the placenta. For each fatty acid, the continuous model and the categorical model were used. The continuous model included the SD score (SDS) as continuous concentrations of fatty acids, in which fatty acid concentrations in maternal erythrocytes were independent variables and TL were dependent variables. The categorical model included quintiles as categorical concentrations of fatty acids. In the categorical model, quintiles were performed as a measure of maternal fatty acid concentration with the middle quintile as a reference to determine the potential non-linearity of the association (41), and the quintiles were independent variables and TL were dependent variables. Then, the gestational age at birth, infant gender, birth weight and length, and maternal age, prepregnancy BMI and gestational weight gain, and paternal age and BMI were included in a multivariate regression analysis based on the categorical model to explore the adjusted contributions of the individual fatty acids. The analysis of the associations between fatty acid concentrations and TL in the cord blood was also performed with fatty acid concentrations as independent variables and TL as dependent variables. Similarly, the associations between methylation fractions of CpG sites in the TERT promoter and TL in the umbilical cord blood and placenta were performed using methylation fractions as independent variables and TL as dependent variables. The associations between maternal PUFAs and the TERT promoter methylation in the umbilical cord blood and placenta were analyzed using fatty acid concentrations as independent variables and average methylation fractions of the TERT promoter as dependent variables. *p* < 0.05 was considered to be statistically significant.

## Results

### Characteristics of Mother–Newborn Pairs

The characteristics of mothers and their newborn babies are shown in Table 1. Average age was 31.96 ± 3.70 years and 33.72 ± 4.62 years for mothers and fathers, respectively. Maternal prepregnancy BMI (21.70 ± 3.01 kg/m^2^) was in the normal range, whereas paternal BMI (25.45 ± 3.67 kg/m^2^) was located in the range of overweight based on Chinese population standards. Almost all mothers during pregnancy were given folic acid supplementation for the purpose of infant neural tube defect prevention. Dietary records showed that 52.19% of mothers received DHA supplementation during pregnancy, who had higher erythrocyte DHA concentrations than those with no DHA supplementation (Supplementary Table 1). The newborns had a mean gestational age of 39.23 ± 1.36 weeks, with a ratio of male to female at 1.03:1. The mean birth weight and length were within the normal range based on the Chinese child growth chart. Maternal age, prepregnancy BMI and parity, paternal age and BMI, and gestational age at birth and birth weight had no relations with TL in the umbilical cord blood or in the placenta. Newborn gender was associated with placenta TL (−0.23 ± 0.09, *p* < 0.05), with a shorter TL in boys than in girls.

**Table 1:** Characteristics of parents and newborns (*n* = 274).

**Characteristic**	**Value**
**Mothers**	
Age (years)	31.96 ± 3.70^1^
Pre-pregnancy BMI (kg/m2)	21.70 ± 3.01
Gestational weight gain (kg)	13.25 ± 4.97
Parity (% nullipara)	56.93
Folic supplementation (%)	96.71
Fish oil supplementation (%)	52.19
**Fathers**	
Age (years)	33.72 ± 4.62
BMI (kg/m2)	25.45 ± 3.67
**Newborns**	
Gestational age (weeks)	39.23 ± 1.36
Gender (% M)	50.73
Weight at birth (g)	3,313.42 ± 403.22
Weight at birth SDS	0.23 ± 1.08
Length at birth (cm)	49.51 ± 1.76
Length at birth SDS	−0.29 ± 0.86
BMI at birth (kg/m2)	13.47 ± 1.10
BMI at birth SDS	0.34 ± 0.89

*$\bar{X}$ ± SD (all such values). BMI, Body Mass Index; SDS, SD Score*.

### Associations of Maternal PUFAs and TL in Umbilical Cord Blood and Placenta

**Figure 2:**
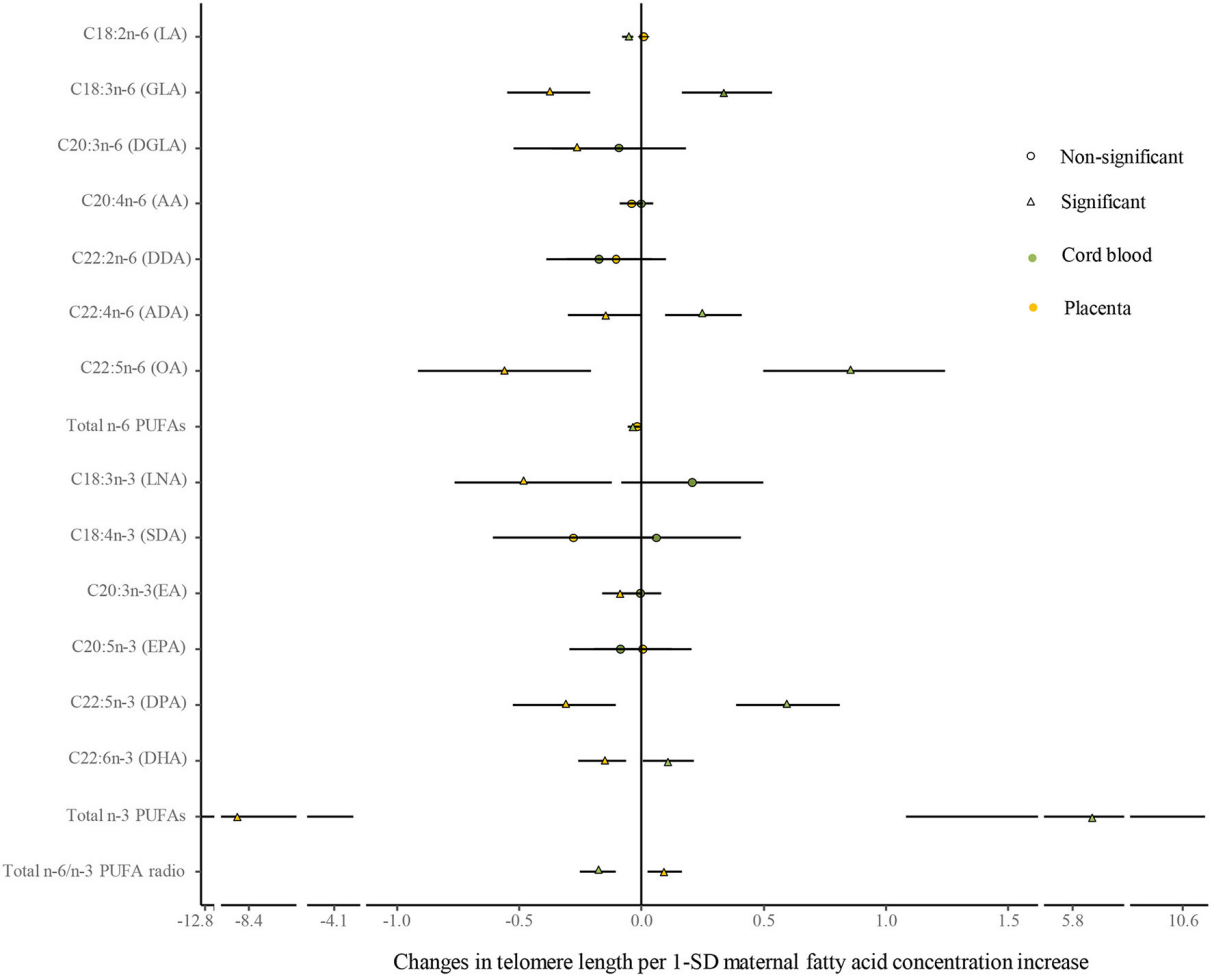
Analysis of the differences in the associations of maternal erythrocyte fatty acids and TL between the cord blood and the placenta by continuous univariate regression. The continuous model is used to include the SD score as continuous concentrations of fatty acids, where fatty acid concentrations in maternal erythrocytes are independent variables and TL are dependent variables. β-coefficients and 95% CI represent changes in TL with 1-SD increase in fatty acid concentration. Circle: non-significant; triangle: significant; color green: the cord blood; color yellow: the placenta.

**Figure 3:**
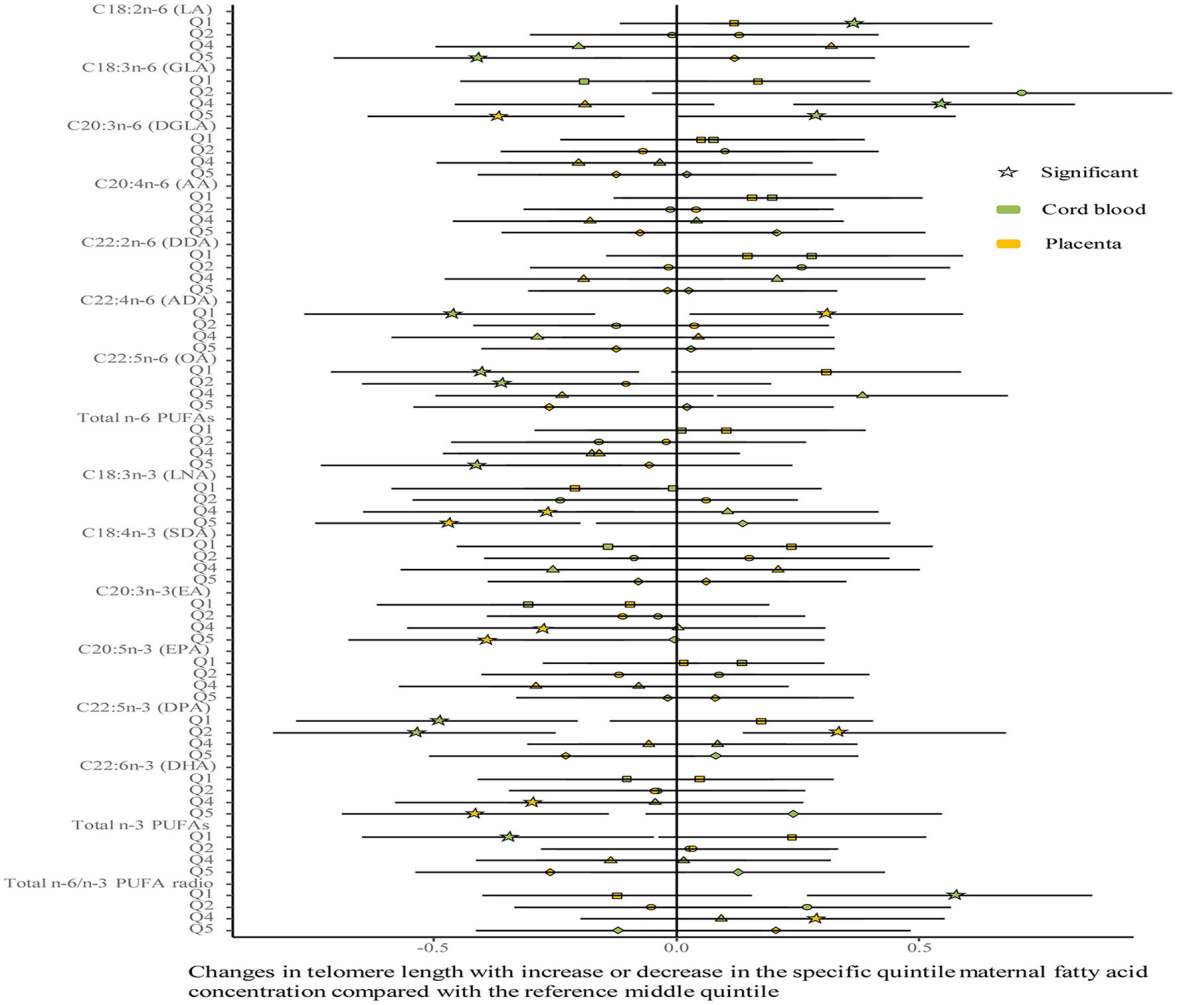
Analysis of differences in the associations of maternal erythrocyte fatty acids and TL between the cord blood and the placenta by categorical multivariate regression. The categorical model is used to include quintiles as categorical concentrations of fatty acids. Quintiles are performed as a measure of maternal fatty acids concentration with the middle quintile as a reference (see Supplementary Table 2) to determine the potential nonlinearity of the association, with the quintiles as independent variables and TL as dependent variables. β-coefficients and 95% CI are adjusted for gestational age at birth, infant sex, birth weight and length, and maternal age, maternal prepregnancy BMI, and gestational weight gain, and paternal age and BMI, and represent differences between TL in the specific quintile and that in the reference middle quintile. Star: significant; color green: the cord blood; color yellow: the placenta.

**Figure 4:**
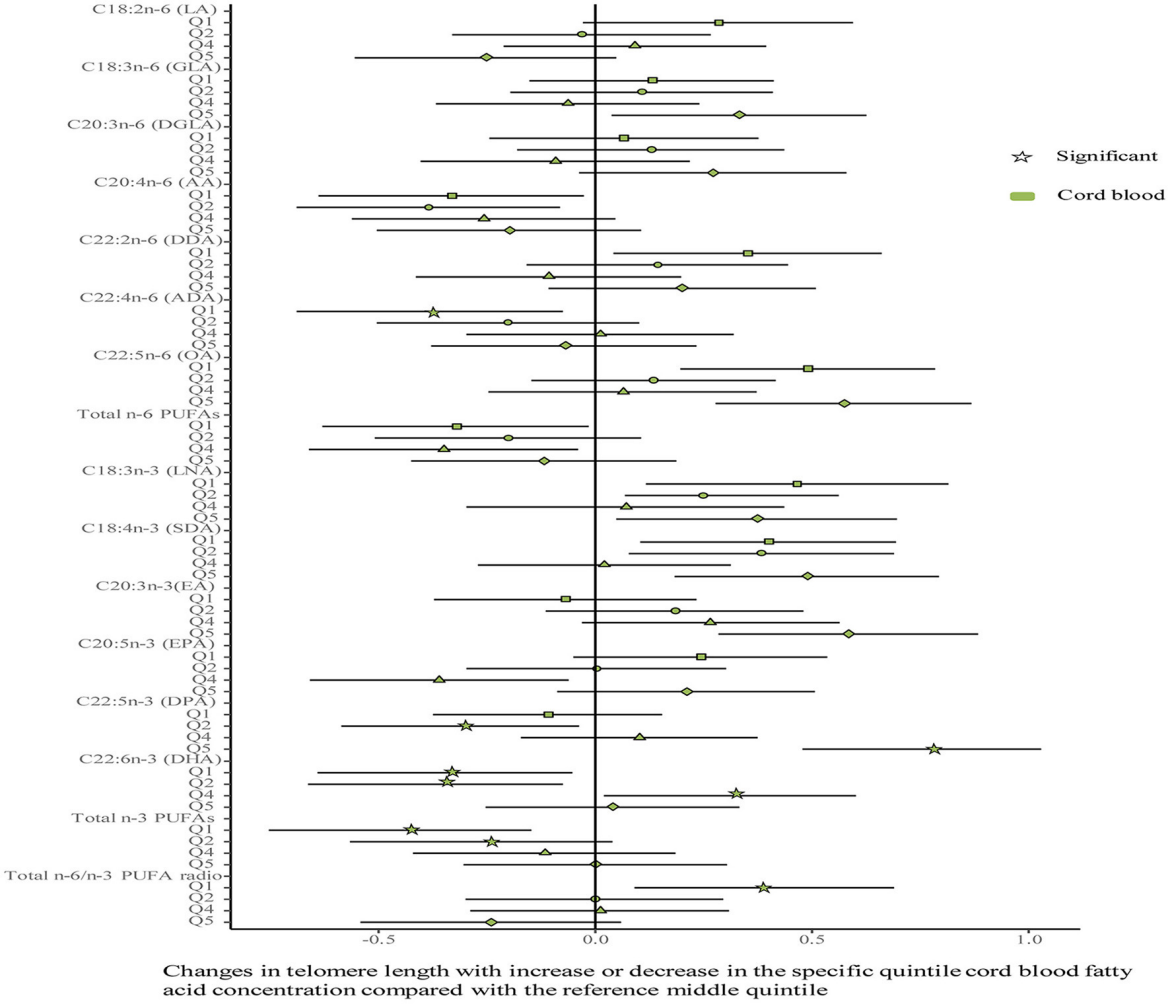
Associations between fatty acids and TL in the cord blood. The categorical model is used to include quintiles as categorical concentrations of fatty acids. Quintiles are performed as a measure of maternal fatty acids concentration with the middle quintile as a reference (see Supplementary Table 4) to determine the potential nonlinearity of the association, with the quintiles as independent variables and TL as dependent variables. β-coefficients and 95% CI are adjusted for gestational age at birth, infant sex, birth weight and length, and maternal age, maternal prepregnancy BMI, and gestational weight gain, and paternal age and BMI, and represent differences between TL in the specific quintile and that in the reference middle quintile. Star: significant; color green: the cord blood.

No differences were shown in TL between the umbilical cord blood (0.99 ± 0.08) and the placenta (0.97 ± 0.59). Maternal erythrocyte n-3 PUFA concentrations with quintile distributions are presented in Supplementary Table 2. The associations between maternal erythrocyte PUFA concentrations and TL in cord blood cells are shown in Figures 2, 3 (Supplementary Table 3). Continuous univariate regression showed that TL was positively related to concentrations of γ-linoleic acid (GLA, C18: 3n-6), adrenic acid (ADA, C22: 4n-6), osbond acid (OA, C22: 5n-6), docosapentaenoci acid (DPA, C22: 5n-3), DHA (C22: 6n-3), and total n-3 PUFAs, but negatively related to linoleic acid (LA, C18: 2n-6), total n-6 PUFAs, and total n-6/n-3 PUFA ratio. Categorical analysis showed these associations existing at the extremes of exposure scale, characterized by associations at both the lowest and highest quintile of LA, the highest quintile of GLA and total n-6 PUFAs, and the lowest quintile of ADA, OA, DPA, total n-3 PUFAs, and n-6/n-3 PUFA ratio. The highest quintile of maternal LA concentration and total n-6 PUFAs shortened TL at 0.39, while the lowest quintile of ADA, OA, DPA, and total n-3 PUFAs shortened TL at 0.36–0.46 in cord blood cells, compared with the middle reference quintile. Unfortunately, no relations between cord blood TL and maternal DHA concentrations were indicated in any quintile. A multivariate analysis showed that the adjustment for factors in infants (gestational age at birth, infant sex, birth weight, and length), mothers (maternal age, prepregnancy BMI, and gestational weight gain), and fathers (paternal age and BMI), did not change the directions of the associations except for DHA. However, based on quintile distributions of PUFAs in cord blood cells (Supplementary Table 4), positive associations between cord blood TL and DHA concentration in cord blood cells remained significant after the adjustment for relevant variables as shown in Figure 4 (Supplementary Table 5).

The associations between maternal erythrocyte PUFA concentrations and TL in the placenta are shown in Figures 2, 3 (Supplementary Table 6). Placenta TL was negatively related to maternal GLA, dihomo-γ-linolenic acid (DGLA, C20: 3n-6), OA, ADA, LNA, eicosatrienoic acid (EA, C20: 3n-3), DPA, DHA, and total n-3 PUFAs, but positively related to n-6/n-3 PUFA ratio with continuous univariate regression. Categorical analysis showed the persisted tendency in associations between placenta TL and fatty acids (exception: DGLA). The highest quintile of GLA, LNA, EA, and DHA shortened placenta TL from 0.36 to 0.45, whereas the lowest quintile of ADA, OA, and total n-3 PUFAs prolonged placenta TL from 0.26 to 0.35, compared with the middle reference quintile. Except for DGLA, OA, and total n-3 PUFAs, the adjustment for relevant factors in infants, mothers, and fathers did not change the directions of the associations using the multivariate analysis.

### Correlative Analysis Between TL and TERT Promoter Methylation in Umbilical Cord Blood and Placenta

**Figure 5:**
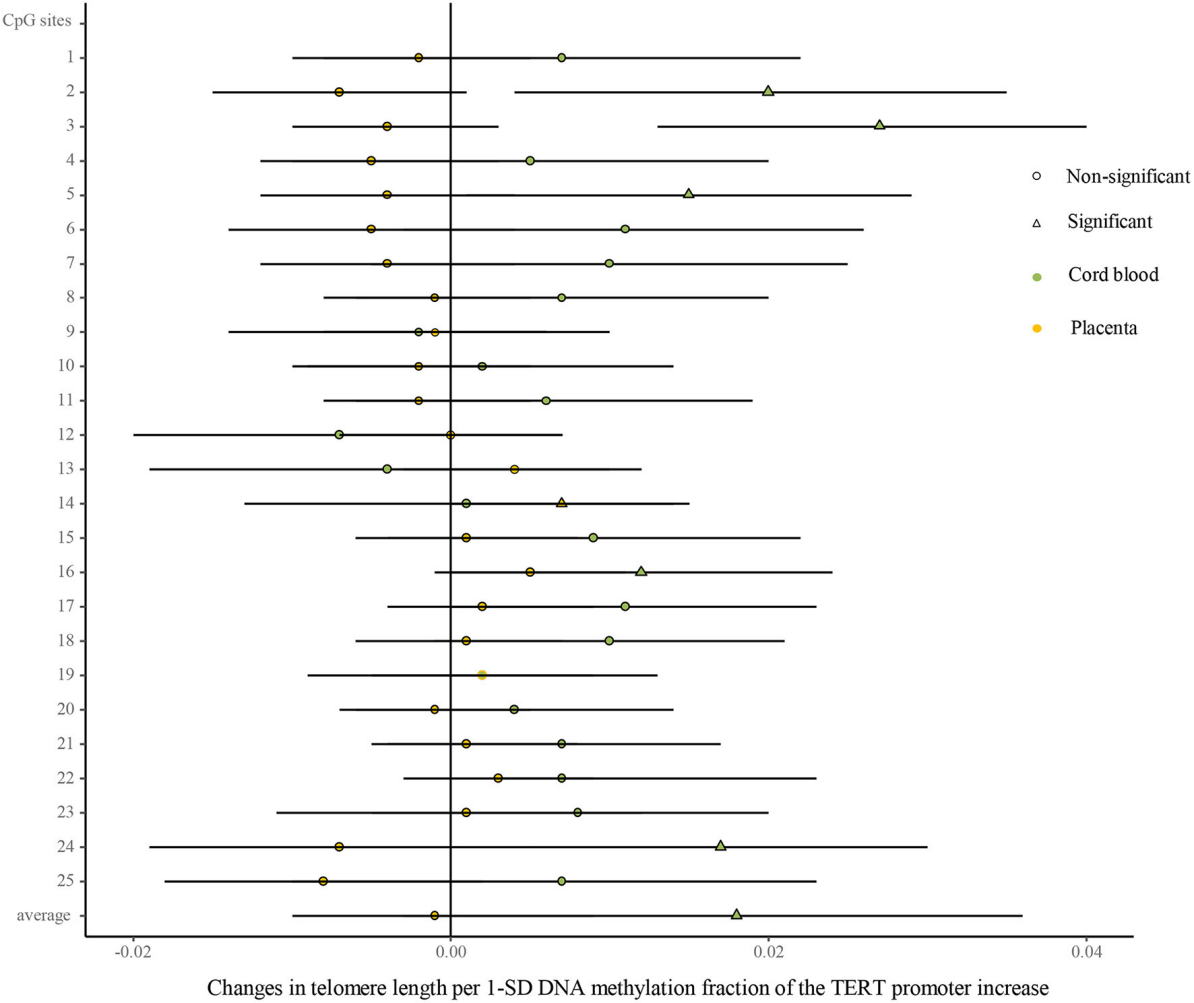
Differences in the associations of methylation fractions of CpG sites in the TERT promoter and TL between the cord blood and the placenta. The continuous model is used to include the SD score as continuous DNA methylation fractions of the TERT promoter, where DNA methylation fractions are independent variables and TL are dependent variables. β-coefficients and 95% CI are adjusted for gestational age at birth, infant sex, birth weight and length, and maternal age, maternal prepregnancy BMI, and gestational weight gain, and paternal age and BMI, and represent changes in TL in the cord blood and the placenta with 1-SD increase in methylation fractions of CpG sites of the TERT promoter. Circle: non-significant; triangle: significant; color green: the cord blood; color yellow: the placenta.

As shown in Figure 5 (Supplementary Table 7), univariate analysis showed that the methylated fractions in 5 of the 25 CpG sites and their average methylation fractions in the TERT promoter were positively correlated with TL in cord blood cells. After the adjustment for factors in infants, mothers, and fathers by multivariate regression, the methylated fractions of the 5 CpG sites remained positive in relation to cord blood TL. In the placenta, the methylated fractions in 2 of the 25 CpG sites were slightly related to TL by the univariate analysis, and after the adjustment for factors in infants, mothers, and fathers by multivariate regression, one CpG site methylation remained slightly positive in relation to placenta TL.

### Correlative Analysis Between Maternal PUFAs and TERT Promoter Methylation in Umbilical Cord Blood and Placenta

**Figure 6:**
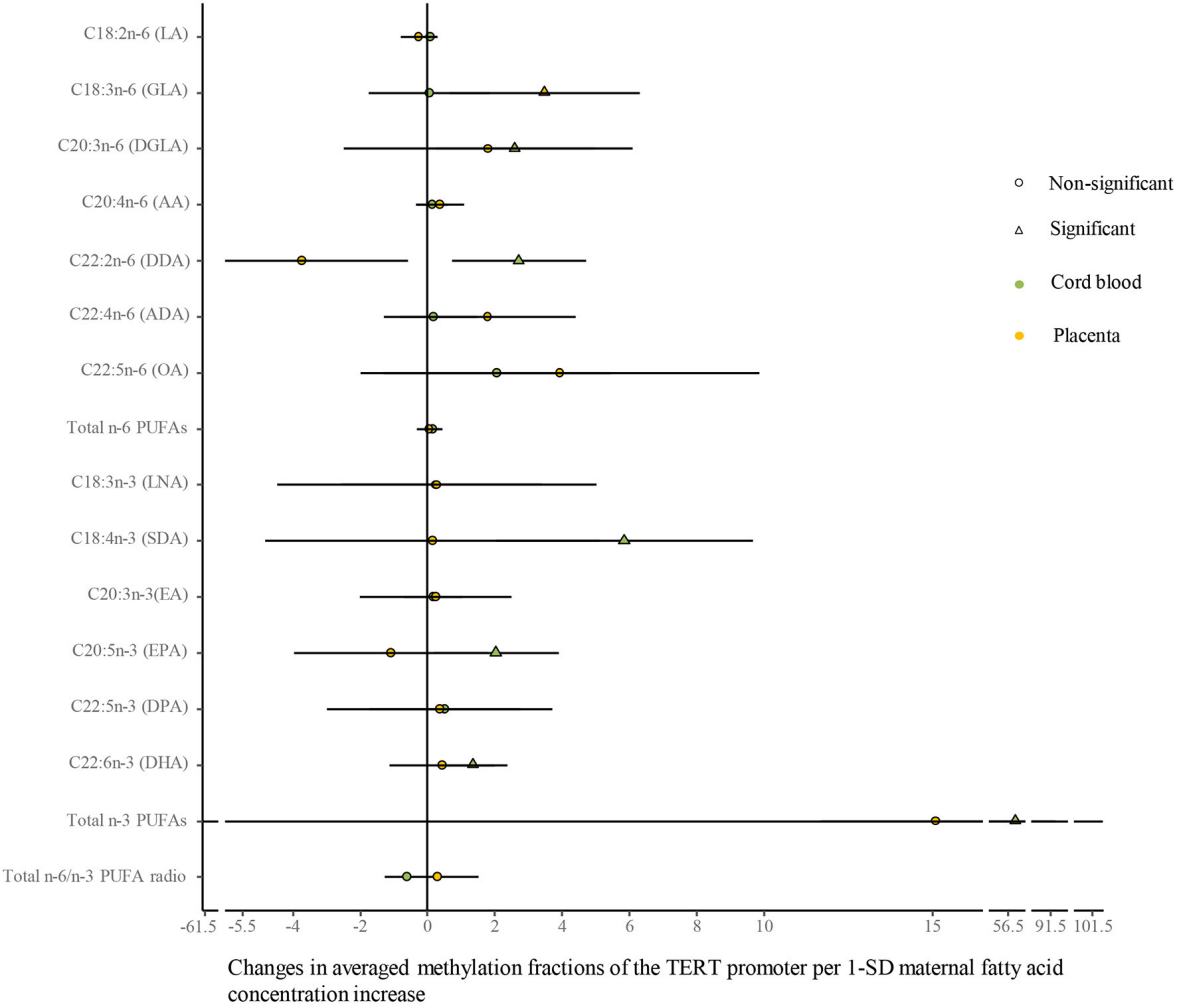
Differences in the associations of maternal fatty acids and average methylation fractions of CpG sites in the TERT promoter between the cord blood and the placenta. The continuous model is used to include the SD score as maternal fatty acid concentrations, where maternal fatty acid concentrations are independent variables and average methylation fractions of CpG sites in the TERT promoter are dependent variables. β-coefficients and 95% CI are adjusted for gestational age at birth, infant sex, birth weight and length, and maternal age, maternal prepregnancy BMI, and gestational weight gain, and paternal age and BMI, and represent the changes in average methylation fractions of CpG sites in the TERT promoter in the cord blood and the placenta with a 1-SD increase in maternal fatty acids. Circle: non-significant; triangle: significant; color green: the cord blood; color yellow: the placenta.

As shown in Figure 6 (Supplementary Table 8), univariate analysis showed that average methylation fractions of the TERT promoter in cord blood cells were positively related to maternal concentrations of DGLA, docosadienoic acid (DDA, C22:2n-6), stearidonic acid (SDA, C18:4n-3), EPA, DHA, and total n-3 PUFAs, and those in the placenta were positively related to maternal GLA concentrations. The adjustment for relevant factors in infants, mothers, and fathers did not change the directions of the associations using the multivariate analysis.

## Discussion

In the current study, after the adjustment for relevant variables, lower concentrations of maternal erythrocyte GLA, ADA, OA, DPA, and total n-3 PUFAs, and higher concentrations of LA, total n-6 PUFAs, and higher n-6/n-3 PUFA ratio were associated with the shortened TL in cord blood cells. Further analysis demonstrated a positive correlation between cord blood TL and DHA concentrations in cord blood cells instead of maternal erythrocytes, implying the effect of maternal erythrocyte n-3 PUFAs on cord blood TL might be associated with increased DHA levels in the fetus. Whereas, in the placenta, higher concentrations of maternal erythrocyte GLA, LNA, EA, and DHA were associated with the shortened TL. So far, the associations of the maternal PUFA status with offspring TL have been reported in only two studies, showing no correlations between maternal n-3 PUFAs and TL in newborns and children (30, 31), which is inconsistent with our findings. Nevertheless, the associations between n-3 PUFAs and TL have been demonstrated in people with specific lifestyle and dietary patterns (39, 42–45), and chronic diseases, including cardiovascular disease and obesity (28, 38, 46, 47). Thus, our findings herein indicate that the poor maternal n-3 PUFA status and/or a higher intake of n-6 PUFAs during pregnancy may shorten TL in the offspring, which is supposed to be a determinant for health and disease susceptibility in later life (7).

Being different from the Paleolithic diet, modern westernized diets are deficient in n-3 PUFAs and have excessive amounts of n-6 PUFAs, resulting in a high n-6/n-3 ratio, which has been shown to promote the pathogenesis of many diseases, including cardiovascular disease, cancer, and inflammatory and autoimmune diseases (28, 48). The underlying mechanisms are dependent on LA- and AA-derived lipid mediators, which enhance inflammation, platelet aggregation, and vasoconstriction, and the hyperactive endocannabinoid system (49). Meanwhile, differential effects of n-6 and n-3 PUFAs on TL have been clarified, showing that the former shortens TL and the latter prolongs TL (50). It has been reported that TL increases with decreasing n-6/n-3 ratios, and thus impacts cell aging, which together with inflammation and oxidative stress represents important pre-disease mechanisms (28). Furthermore, our previous study indicates that lower body DHA content and a higher AA/DHA ratio are associated with the shortened TL in child obesity (38).

Interestingly, we found that maternal LA, total n-6 PUFAs, and n-6/n-3 PUFA ratio were negatively associated with cord blood TL, whereas GLA, ADA, and OA belonging to n-6 series were positively associated. The reason for this to happen may be involved in their effects to inhibit inflammation, which is a risk factor for TL shortening. For example, GLA is converted into DGLA and forms a substrate for cyclooxygenase enzyme that fights inflammation, being in contrast to LA and AA, which tend to promote inflammation (51). Also, it is found that GLA together with ascorbate improves skeletal ossification in the offspring of diabetic rats (52). ADA potently inhibits the formation of leukotriene B4, which correlates with a reduction of its precursor AA, and thus could play a role in the resolution of inflammation (53).

Another interesting finding was the negative associations of maternal n-3 PUFAs (LNA, EA, and DHA) and GLA with TL in the placenta, which was in contrast to the results in the cord blood. This might be attributable to the differences in lifespan, functions, and sensitivity to maternal nutrition between the placenta and cord blood (17, 33). In comparison with the cord blood cells that are at early hematopoiesis and abundant in telomerase-expressing stem cells, placenta cells undergo cellular senescence such that they may have shortened TL (17). As the pregnancy progresses, telomerase activity in the placenta declines, resulting in shortened TL across gestation with the shortest telomeres at term (54, 55), probably due to the increasing production of mediators, particularly pro-inflammatory cytokines, neutrophil recruitment chemokines, and arachidonic acid metabolites of chorioamniotic cells in the third trimester of pregnancy (56). However, few studies indicate a discrepancy in the results of TL in the cord blood compared to the placenta (19, 57), and we found no differences in TL between the cord blood and the placenta in the current study. Meanwhile, TL in the placenta may have different responses from cord blood cells to environmental stimuli, including maternal nutritional status (17, 19, 33). Recently, Vahter et al. have reported that maternal BMI, body fat percentage, and vitamin B_12_ are inversely associated with placental TL, whereas 25 (OH) D_3_ is positively associated, and no associations were observed with cord blood TL (19).

The TERT expression represents the rate-limiting step in telomerase expression and activity and plays a key role in maintaining TL during cellular aging, immortalization, and transformation (58). DNA methylation, a kind of epigenetic modification, is principally involved in regulating gene expression, and DNA hypermethylation of a gene promoter is generally associated with gene silencing (59, 60). Inconsistently, we found that DNA methylation fractions of the TERT promoter had a positive association with TL in cord blood cells, implying the upregulated TERT expression and a high telomerase activity. The data presented here are in keeping with most reports that the TERT promoter hypermethylation is positively associated with the TERT expression and telomerase activity in cancers (61, 62). This may be explained by the specific characteristics of the cord blood in which high-potentially differentiated cells, hematopoietic stem cells, are abundant (34). Furthermore, we found that maternal concentrations of DHA and total n-3 PUFAs were positively associated with DNA methylation fractions of the TERT promoter in cord blood cells, and thus influencing TL. This suggests that the mechanisms by which n-3 PUFAs affect TL may reside in both their functions in epigenetic modification and their anti-inflammatory or anti-oxidative stress effects (27–29). To note, no significant associations of the TERT promoter methylation with TL in the placenta were found in our study, indicating that TL in the placenta might be primarily influenced by inflammation or oxidation instead of the TERT methylation and associated telomerase activity, which are in higher levels in late pregnancy (56). Still, no associations between maternal DHA and total n-3 PUFAs and the TERT promoter methylation were found herein, indicating that the effects of maternal n-3 PUFAs on placenta TL may depend on their regulatory roles in inflammation or metabolic processes (63), rather than their functions in epigenetic modifications.

Our data suggest that a higher intake of n-3 PUFAs during pregnancy may help to maintain TL in the offspring, which is beneficial for long-term health. Some limitations of our study should be addressed. Previous studies have demonstrated the associations between TL and chronic diseases, including cardiovascular diseases and obesity. Unfortunately, we only examined TL in the cord blood cells and the placenta with no data on TL after birth available, and thus its association with chronic diseases in later life could not be investigated in the current study. Meanwhile, we did not determine single-nucleotide polymorphisms in the fatty acid desaturase, which has strong effects on levels of long-chain PUFAs. Thus, this genetic factor should be included in analyzing the association of PUFAs with TL in the future.

## Conclusion

In summary, low concentrations of maternal n-3 PUFAs and some of n-6 PUFAs (GLA, ADA, and OA), and high concentrations of LA and high n-6/n-3 PUFA ratio were associated with the shortened TL in cord blood cells; low concentrations of cord blood DHA were related to the shortened cord blood TL. Whereas, in the placenta, higher concentrations of either maternal n-3 PUFAs (LNA, EA, and DHA) or GLA were associated with the shortened TL. Furthermore, maternal DHA and total n-3 PUFAs had a positive association with the TERT promoter DNA methylation in the cord blood instead of the placenta. Therefore, maternal PUFAs are closely correlated to infant TL and the TERT promoter methylation. Different effects of maternal n-3 PUFAs on TL and the TERT promoter methylation between the cord blood and the placenta are suggested.

## Data Availability Statement

The original contributions presented in the study are included in the article/Supplementary Materials, further inquiries can be directed to the corresponding author/s.

## Ethics Statement

The studies involving human participants were reviewed and approved by Institutional Review Board and Committee on Human Research at Fu Xing Hospital, Capital Medical University. Written informed consent to participate in this study was provided by the participants' legal guardian/next of kin.

## Author Contributions

XL performed the experiment, analyzed the data, and prepared the manuscript. QS performed the experiment. XF participated in designing the research and analyzed leukocyte TL. HC, NC, and YZ performed questionnaires and sample collection. KQ designed the research and revised the manuscript. All authors contributed to the article and approved the submitted version.

## Funding

This work was supported by the Research Funds of Reform and Development Budget from Beijing Municipal Science and Technology Commission (2020-bjsekyjs to KQ), the National Key R&D Program of China (KQ, 2016YFC1305201), and Beijing Xicheng District Talent Project - top team (202048). The funders had no role in study design, data collection and analysis, interpretation of data, decision to publish, or preparation of the manuscript.

## Conflict of Interest

The authors declare that the research was conducted in the absence of any commercial or financial relationships that could be construed as a potential conflict of interest.

## Publisher's Note

All claims expressed in this article are solely those of the authors and do not necessarily represent those of their affiliated organizations, or those of the publisher, the editors and the reviewers. Any product that may be evaluated in this article, or claim that may be made by its manufacturer, is not guaranteed or endorsed by the publisher.

## Abbreviations

BMI: body mass index
PUFAs: polyunsaturated fatty acids
TERT: telomerase reverse transcriptase
TL: telomere length
GLA: γ-linoleic acid
ADA: adrenic acid
DPA: docosapentaenoci acid
DHA: docosahexaenoic acid
EPA: eicosapentaenoic acid
DoHaD: developmental origins of health and disease
LA: linoleic acid
AA: arachidonic acid
LNA: α-linolenic acid
DGLA: dihomo-γ-linolenic acid
SDA: stearidonic acid
OA: osbond acid
EA: eicosatrienoic acid
DDA: docosadienoic acid
SDS: SD score.
