# Supplementary material for: Associations of Maternal Polyunsaturated Fatty Acids With Telomere Length in the Cord Blood and Placenta in Chinese Population
Source: Front Nutr. 2022 Jan 28;8:779306. doi: 10.3389/fnut.2021.779306 (PMC8831827; doi:10.3389/fnut.2021.779306)
Supplement: Supplementary file 1 [file Table_1.docx]

Supplementary Material

**Table 1. Changes in concentration of PUFAs by fish oil supplementation in maternal erythrocytes (n = 274).**

|  | Fish oil supplement^2^ | |  |  |
| --- | --- | --- | --- | --- |
| Fatty acids^1^ | Yes (n = 138) | No (n = 136) | t | P |
| **Total n-6 PUFAs** | 26.61±4.33 | 26.87±4.59 | 0.486 | 0.627 |
| C18:2n-6 (LA) | 13.5±3.95 | 13.32±4.14 | 0.418 | 0.676 |
| C18:3n-6 (GLA) | 0.49±0.53 | 0.45±0.48 | 0.728 | 0.467 |
| C20:3n-6 (DGLA) | 1.19±0.38 | 1.23±0.32 | 0.792 | 0.429 |
| C20:4n-6 (AA) | 8.53±1.91 | 8.78±1.92 | 1.068 | 0.286 |
| C22:2n-6 (DDA) | 1.55±0.40 | 1.56±0.49 | 0.228 | 0.820 |
| C22:4n-6 (ADA) | 1.09±0.63^3^ | 1.30±0.55 | 3.011 | 0.003 |
| C22:5n-6 (OA) | 0.26±0.25 | 0.27±0.25 | 0.143 | 0.887 |
| **Total n-3 PUFAs** | 6.79±1.95^3^ | 6.17±1.96 | 2.640 | 0.009 |
| C18:3n-3 (LNA) | 0.34±0.37 | 0.32±0.28 | 0.346 | 0.730 |
| C18:4n-3 (SDA) | 0.96±0.32 | 0.93±0.22 | 0.714 | 0.476 |
| C20:3n-3 (EA) | 0.90±1.23 | 0.82±1.01 | 0.583 | 0.561 |
| C20:5n-3 (EPA) | 1.71±0.44 | 1.63±0.48 | 1.322 | 0.187 |
| C22:5n-3 (DPA) | 0.54±0.45 | 0.55±0.40 | 0.142 | 0.887 |
| C22:6n-3 (DHA) | 2.45±0.95^3^ | 2.02±0.80 | 4.121 | 0.000 |
| **Total n-6/n-3 PUFAs** | 4.11±1.14^3^ | 4.59±1.33 | 3.216 | 0.001 |

^1^ Relative concentrations (percent of total fatty acids)

^2^‾X±SD(all such values)

^3^‾P <0.05.

**Table 2. Concentrations of** **PUFAs by quintile(Q) in** **maternal erythrocytes (n = 274).**

| Fatty acids^1^ | | Value | Q1 | | Q2 | Q3 | Q4 | | Q5 | |
| --- | --- | --- | --- | --- | --- | --- | --- | --- | --- | --- |
| **Total n-6 PUFAs** | 26.74±4.46 | | ≤23.47 | 23.47-26.01 | | 26.01-27.86 | | 27.86-30.32 | | >30.32 |
| C18:2n-6 (LA) | 13.43±4.04 | | ≤10.76 | 10.76-11.84 | | 11.84-13.13 | | 13.13-15.66 | | >15.66 |
| C18:3n-6 (GLA) | 0.47±0.50 | | 0.00 | 0.00-0.26 | | 0.26-0.45 | | 0.45-0.94 | | >0.94 |
| C20:3n-6 (DGLA) | 1.21±0.35 | | ≤0.95 | 0.95-1.08 | | 1.08-1.22 | | 1.22-1.47 | | >1.47 |
| C20:4n-6 (AA) | 8.65±1.92 | | ≤7.24 | 7.24-8.36 | | 8.36-9.23 | | 9.23-10.09 | | >10.09 |
| C22:2n-6 (DDA) | 1.55±0.45 | | ≤1.18 | 1.18-1.40 | | 1.40-1.58 | | 1.58-1.82 | | >1.82 |
| C22:4n-6 (ADA) | 1.19±0.60 | | ≤0.76 | 0.76-1.17 | | 1.17-1.35 | | 1.35-1.64 | | >1.64 |
| C22:5n-6 (OA) | 0.26±0.25 | | ≤0.05 | 0.05-0.09 | | 0.09-0.34 | | 0.34-0.51 | | >0.51 |
| **Total n-3 PUFAs** | 6.48±1.98 | | ≤4.74 | 4.74-5.89 | | 5.89-6.89 | | 6.89-8.06 | | >8.06 |
| C18:3n-3 (LNA) | 0.33±0.33 | | ≤0.03 | 0.03-0.12 | | 0.12-0.34 | | 0.34-0.59 | | >0.59 |
| C18:4n-3 (SDA) | 0.94±0.28 | | ≤0.76 | 0.76-0.85 | | 0.85-0.96 | | 0.96-1.09 | | >1.09 |
| C20:3n-3  (EA) | 0.86±1.13 | | ≤0.47 | 0.47-0.67 | | 0.67-0.82 | | 0.82-1.01 | | >1.01 |
| C20:5n-3 (EPA) | 1.67±0.46 | | ≤1.28 | 1.28-1.47 | | 1.47-1.70 | | 1.70-1.97 | | >1.97 |
| C22:5n-3 (DPA) | 0.54±0.42 | | ≤0.18 | 0.18-0.26 | | 0.26-0.66 | | 0.66-0.88 | | >0.88 |
| C22:6n-3 (DHA) | 2.24±0.91 | | ≤1.46 | 1.46-1.99 | | 1.99-2.40 | | 2.40-3.00 | | >3.00 |
| **Total n-6/n-3 PUFA radio** | 4.35±1.26 | | ≤3.37 | 3.37-3.85 | | 3.85-4.48 | | 4.48-5.18 | | >5.18 |

^1^ Relative concentrations (percent of total fatty acids)

^2^‾X±SD(all such values)

**Table 3. Associations between fatty acids in maternal** **erythrocytes and TL in the cord blood (n=274).**

|  | | n | TL^1^ | | Univariate model^2^ | | Multivariate model^2,4,5^ |
| --- | --- | --- | --- | --- | --- | --- | --- |
| Fatty acids | |  |  |  | Continuous^3^ | Categorical^4^ |  |
| C18:2n-6 (LA) | | |  | | -0.057±0.012^6^ |  |  |
| Q1 | | 54 | 1.38±1.00 | |  | 0.359±0.148^6^ | 0.357±0.149^6^ |
| Q2 | | 55 | 1.01±0.78 | |  | 0.026±0.148 | -0.008±0.149 |
| Q3 | | 55 | 1.00±0.77 | |  | Reference | Reference |
| Q4 | | 55 | 0.96±0.69 | |  | -0.179±0.146 | -0.201±0.149 |
| Q5 | | 55 | 0.71±0.56 | |  | -0.385±0.147^6^ | -0.409±0.150^6^ |
| C18:3n-6 (GLA) | | | | | 0.349±0.094^6^ |  |  |
| Q1 | | 106 | 0.70±0.66 | |  | -0.187±0.128 | -0.190±0.129 |
| Q2 | | 4 | 1.41±1.09 | |  | 0.695±0.393 | 0.710±0.395 |
| Q3 | | 55 | 0.95±0.71 | |  | Reference | Reference |
| Q4 | | 54 | 1.41±0.82 | |  | 0.514±0.146^6^ | 0.531±0.147^6^ |
| Q5 | | 55 | 0.98±0.69 | |  | 0.274±0.145^6^ | 0.288±0.146^6^ |
| C20:3n-6 (DGLA) | | | | | -0.092±0.140 |  |  |
| Q1 | | 56 | 1.03±0.79 | |  | 0.075±0.154 | 0.076±0.158 |
| Q2 | | 53 | 1.03±0.84 | |  | 0.079±0.156 | 0.100±0.160 |
| Q3 | | 56 | 1.01±0.78 | |  | Reference | Reference |
| Q4 | | 55 | 0.86±0.75 | |  | -0.038±0.153 | -0.034±0.159 |
| Q5 | | 54 | 1.00±0.75 | |  | 0.025±0.154 | 0.022±0.156 |
| C20:4n-6 (AA) | | |  | | -0.001±0.025 |  |  |
| Q1 | | 54 | 1.09±0.84 | |  | 0.212±0.154 | 0.197±0.157 |
| Q2 | | 55 | 0.89±0.75 | |  | -0.011±0.152 | -0.012±0.154 |
| Q3 | | 55 | 0.89±0.76 | |  | Reference | Reference |
| Q4 | | 55 | 0.95±0.77 | |  | 0.051±0.152 | 0.041±0.154 |
| Q5 | | 55 | 1.12±0.86 | |  | 0.223±0.152 | 0.207±0.156 |
| C22:2n-6 (DDA) | | | |  | -0.174±0.109 |  |  |
| Q1 | | 55 | 1.12±0.85 | |  | 0.299±0.154^6^ | 0.279±0.158^6^ |
| Q2 | | 56 | 1.06±0.67 | |  | 0.242±0.153 | 0.257±0.155 |
| Q3 | | 53 | 0.82±0.65 | |  | Reference | Reference |
| Q4 | | 56 | 1.07±0.98 | |  | 0.226±0.151 | 0.207±0.156 |
| Q5 | | 54 | 0.86±0.84 | |  | 0.030±0.153 | 0.025±0.156 |
| C22:4n-6 (ADA) | | | | | 0.253±0.080^6^ |  |  |
| Q1 | | 54 | 0.69±0.66 | |  | -0.440±0.150^6^ | -0.467±0.152^6^ |
| Q2 | | 57 | 1.03±0.70 | |  | -0.104±0.149 | -0.123±0.150 |
| Q3 | | 54 | 1.04±0.77 | |  | Reference | Reference |
| Q4 | | 54 | 0.86±0.75 | |  | -0.275±0.149 | -0.286±0.152 |
| Q5 | | 55 | 1.20±0.89 | |  | 0.062±0.148 | 0.030±0.150 |
| C22:5n-6 (OA) | | |  | | 0.870±0.188^6^ |  |  |
| Q1 | | 54 | 0.68±0.62 | |  | -0.377±0.157^6^ | -0.393±0.161^6^ |
| Q2 | | 64 | 0.71±0.72 | |  | -0.351±0.143^6^ | -0.363±0.144^6^ |
| Q3 | | 46 | 1.06±0.76 | |  | Reference | Reference |
| Q4 | | 56 | 1.44±0.93 | |  | 0.384±0.151^6^ | 0.383±0.152^6^ |
| Q5 | | 54 | 1.08±0.72 | |  | 0.022±0.152 | 0.022±0.153 |
| Total n-6 PUFAs | | | | | -0.037±0.011^6^ |  |  |
| Q1 | | 56 | 1.15±0.88 | |  | 0.038±0.151 | 0.010±0.154 |
| Q2 | | 54 | 0.97±0.75 | |  | -0.142±0.152 | -0.160±0.155 |
| Q3 | | 55 | 1.11±0.77 | |  | Reference | Reference |
| Q4 | | 55 | 0.98±0.90 | |  | -0.130±0.152 | -0.174±0.155 |
| Q5 | | 54 | 0.73±0.65 | |  | -0.384±0.152^6^ | -0.421±0.158^6^ |
| C18:3n-3 (LNA) | | | | | 0.208±0.148 |  |  |
| Q1 | | 53 | 1.02±0.87 | |  | 0.032±0.154 | -0.006±0.156 |
| Q2 | | 57 | 0.76±0.67 | |  | -0.221±0.151 | -0.239±0.153 |
| Q3 | | 55 | 0.98±0.70 | |  | Reference | Reference |
| Q4 | | 54 | 1.08±0.90 | |  | 0.095±0.153 | 0.106±0.157 |
| Q5 | | 55 | 1.11±0.85 | |  | 0.126±0.153 | 0.137±0.154 |
| C18:4n-3 (SDA) | | | | | 0.062±0.175 |  |  |
| Q1 | | 59 | 0.94±0.67 | |  | -0.142±0.155 | -0.141±0.158 |
| Q2 | | 59 | 1.07±0.90 | |  | -0.091±0.155 | -0.087±0.157 |
| Q3 | | 47 | 1.02±0.90 | |  | Reference | Reference |
| Q4 | | 55 | 0.85±0.68 | |  | -0.246±0.157 | -0.254±.0160 |
| Q5 | | 54 | 1.06±0.83 | |  | -0.049±0.154 | -0.078±0.157 |
| C20:3n-3 (EA) | | |  | | -0.004±0.043 |  |  |
| Q1 | | 54 | 0.77±0.70 | |  | -0.291±0.155^6^ | -0.305±0.158^6^ |
| Q2 | | 56 | 0.99±0.74 | |  | -0.030±0.151 | -0.038±0.154 |
| Q3 | | 54 | 1.05±0.82 | |  | Reference | Reference |
| Q4 | | 57 | 1.06±0.80 | |  | 0.021±0.151 | 0.004±0.154 |
| Q5 | | 53 | 1.05±0.84 | |  | 0.001±0.153 | -0.004±0.157 |
| C20:5n-3 (EPA) | | | | | -0.087±0.106 |  |  |
| Q1 | | 54 | 1.07±0.81 | |  | 0.128±0.157 | 0.135±0.160 |
| Q2 | | 55 | 1.05±0.74 | |  | 0.089±0.153 | 0.088±0.157 |
| Q3 | | 55 | 0.98±0.78 | |  | Reference | Reference |
| Q4 | | 55 | 0.86±0.84 | |  | -0.064±0.155 | -0.078±0.157 |
| Q5 | | 55 | 0.97±0.87 | |  | -0.002±0.154 | -0.018±0.158 |
| C22:5n-3 (DPA) | | | | | 0.593±0.110^6^ |  |  |
| Q1 | | 55 | 0.69±0.68 | |  | -0.456±0.145^6^ | -0.493±0.147^6^ |
| Q2 | | 56 | 0.63±0.47 | |  | -0.507±0.144^6^ | -0.539±0.147^6^ |
| Q3 | | 55 | 1.14±0.85 | |  | Reference | Reference |
| Q4 | | 57 | 1.25±0.87 | |  | 0.106±0.144 | 0.085±0.146 |
| Q5 | | 51 | 1.24±0.87 | |  | 0.102±0.148 | 0.081±0.149 |
| C22:6n-3 (DHA) | | | | | 0.110±0.053^6^ |  |  |
| Q1 | | 54 | 0.87±0.73 | |  | -0.117±0.153 | -0.102±0.155 |
| Q2 | | 55 | 1.01±0.75 | |  | -0.066±0.152 | -0.039±0.155 |
| Q3 | | 56 | 1.07±0.81 | |  | Reference | Reference |
| Q4 | | 54 | 0.88±0.78 | |  | -0.056±0.153 | -0.043±0.155 |
| Q5 | | 55 | 1.22±0.97 | |  | 0.231±0.152 | 0.241±0.155 |
| Total n-3 PUFAs | | | | | 5.886±2.440^6^ |  |  |
| Q1 | | 56 | 0.68±0.48 | |  | -0.361±0.150^6^ | -0.347±0.152^6^ |
| Q2 | | 54 | 1.05±0.90 | |  | 0.010±0.152 | 0.028±0.155 |
| Q3 | | 55 | 1.04±0.75 | |  | Reference | Reference |
| Q4 | | 54 | 1.03±0.80 | |  | -0.006±0.152 | 0.015±0.154 |
| Q5 | | 55 | 1.15±0.96 | |  | 0.118±0.151 | 0.127±0.154 |
| Total n-6/n-3PUFA radio | | | | | -0.178±0.037^6^ |  |  |
| Q1 | 55 | | 1.39±0.97 | |  | 0.563±0.147^6^ | 0.563±0.149^6^ |
| Q2 | 53 | | 1.08±0.84 | |  | 0.253±0.148 | 0.269±0.150 |
| Q3 | 56 | | 0.83±0.70 | |  | Reference | Reference |
| Q4 | 56 | | 0.93±0.69 | |  | 0.101±0.146 | 0.093±0.148 |
| Q5 | 54 | | 0.70±0.62 | |  | -0.126±0.148 | -0.120±0.149 |

^1^ All values are ‾X±SD.

^2^ All values are B±SE. B means β-coefficients from the regression model.

^3^ Continuous model with the SD score as fatty acid concentrations. B±SE is the unstandardized regression coefficient, representing changes in TL with 1-SD increase in fatty acid concentration.

^4^ Categorical model with quintiles as fatty acid concentrations. B±SE is the unstandardized regression coefficient, representing differences between TL in the specific quintile and that in the reference Q3 quintile.

^5^ Adjusted for gestational age at birth, infant sex, birth weight and length, and maternal age, maternal pre-pregnancy BMI, and gestational weight gain, and paternal age and BMI.

^6^ P <0.05.

**Table 4. Concentration of PUFAs by quintile(Q) in the cord blood (n = 274).**

| Fatty acids^1^ | Value | Q1 | Q2 | Q3 | Q4 | Q5 |
| --- | --- | --- | --- | --- | --- | --- |
| **Total** **n-6 PUFAs** | 23.91±3.76 | ≤20.79 | 20.79-22.72 | 22.72-24.58 | 24.58-27.59 | >27.59 |
| C18:2n-6 (LA) | 4.73±1.55 | ≤3.73 | 3.73-4.21 | 4.21-4.64 | 4.64-5.39 | >5.39 |
| C18:3n-6 (GLA) | 0.51±0.36 | ≤0.26 | 0.26-0.40 | 0.41-0.52 | 0.52-0.68 | >0.68 |
| C20:3n-6(DGLA) | 2.08±0.51 | ≤1.65 | 1.65-1.88 | 1.88-2.15 | 2.15-2.43 | >2.43 |
| C20:4n-6 (AA) | 12.73±2.29 | ≤10.77 | 10.77-12.10 | 12.10-13.21 | 13.21-14.79 | >14.79 |
| C22:2n-6 (DDA) | 1.65±0.73 | ≤1.11 | 1.11-1.30 | 1.30-1.54 | 1.54-2.31 | >2.31 |
| C22:4n-6(ADA) | 1.91±0.84 | ≤1.43 | 1.43-1.75 | 1.75-2.01 | 2.01-2.46 | >2.46 |
| C22:5n-6 (OA) | 0.30±0.33 | ≤0.05 | 0.05-0.11 | 0.11-0.15 | 0.15-0.64 | >0.64 |
| **Total n-3 PUFAs** | 7.08±2.70 | ≤5.28 | 5.28-6.02 | 6.02-6.78 | 6.78-9.25 | >9.25 |
| C18:3n-3 (LNA) | 0.08±0.09 | ≤0.05 | 0.05-0.07 | 0.07-0.08 | 0.08-0.09 | >0.09 |
| C18:4n-3 (SDA) | 0.81±0.25 | ≤0.67 | 0.67-0.76 | 0.76-0.89 | 0.89-1.00 | >1.00 |
| C20:3n-3(EA) | 1.20±1.50 | ≤0.70 | 0.70-0.98 | 0.98-1.10 | 1.10-1.38 | >1.38 |
| C20:5n-3 (EPA) | 2.37±1.03 | ≤1.56 | 1.56-1.89 | 1.89-2.21 | 2.21-3.27 | >3.27 |
| C22:5n-3 (DPA) | 0.18±0.25 | ≤0.06 | 0.06-0.11 | 0.11-0.20 | 0.20-0.24 | >0.24 |
| C22:6n-3(DHA) | 2.60±1.22 | ≤1.60 | 1.60-2.08 | 2.08-2.60 | 2.60-3.61 | >3.61 |
| **Total n-6: n-3 PUFAs** | 3.58±1.10 | ≤2.94 | 2.94-3.34 | 3.34-3.65 | 3.65-4.04 | >4.04 |

^1^ Relative concentrations (percent of total fatty acids)

^2^‾X±SD(all such values)

**Table 5. Associations between fatty acids and TL in the cord blood (n=274).**

|  | | n | TL^1^ | | Univariate model^2^ | | Multivariate model^2,4,5^ |
| --- | --- | --- | --- | --- | --- | --- | --- |
| Fatty acids | |  |  |  | Continuous^3^ | Categorical^4^ |  |
| C18:2n-6 (LA) | | |  | | -0.087±0.031^6^ |  |  |
| Q1 | | 51 | 1.27±0.90 | |  | 0.302±0.155^6^ | 0.284±0.158 |
| Q2 | | 60 | 0.95±0.86 | |  | -0.020±0.149 | -0.032±0.152 |
| Q3 | | 53 | 0.97±0.90 | |  | Reference | Reference |
| Q4 | | 54 | 1.06±0.83 | |  | 0.093±0.153 | 0.091±0.154 |
| Q5 | | 56 | 0.71±0.49 | |  | -0.257±0.151 | -0.253±0.153 |
| C18:3n-6 (GLA) | | | | | 0.219±0.136 |  |  |
| Q1 | | 65 | 1.00±0.98 | |  | 0.105±0.139 | 0.130±0.143 |
| Q2 | | 44 | 0.98±0.82 | |  | 0.090±0.152 | 0.106±0.154 |
| Q3 | | 62 | 0.90±0.74 | |  | Reference | Reference |
| Q4 | | 49 | 0.82±0.58 | |  | -0.071±0.152 | -0.065±0.155 |
| Q5 | | 54 | 1.22±0.77 | |  | 0.329±0.148^6^ | 0.331±0.149^6^ |
| C20:3n-6 (DGLA) | | | | | -0.016±0.095 |  |  |
| Q1 | | 55 | 0.99±0.76 | |  | 0.065±0.154 | 0.065±0.158 |
| Q2 | | 56 | 1.03±0.65 | |  | 0.110±0.153 | 0.128±0.157 |
| Q3 | | 53 | 0.92±0.85 | |  | Reference | Reference |
| Q4 | | 56 | 0.82±0.65 | |  | -0.109±0.153 | -0.093±0.158 |
| Q5 | | 54 | 1.18±1.04 | |  | 0.255±0.155 | 0.271±0.157 |
| C20:4n-6 (AA) | | |  | | 0.006±0.021 |  |  |
| Q1 | | 54 | 0.91±0.64 | |  | -0.311±0.154^6^ | -0.333±0.156^6^ |
| Q2 | | 56 | 0.85±0.70 | |  | -0.364±0.153^6^ | -0.386±0.154^6^ |
| Q3 | | 54 | 1.22±0.83 | |  | Reference | Reference |
| Q4 | | 56 | 0.96±0.75 | |  | -0.259±0.153 | -0.259±0.154 |
| Q5 | | 54 | 1.01±1.02 | |  | -0.207±0.154 | -0.199±0.155 |
| C22:2n-6 (DDA) | | | |  | -0.010±0.067 |  |  |
| Q1 | | 53 | 1.22±0.87 | |  | 0.347±0.156^6^ | 0.351±0.157^6^ |
| Q2 | | 59 | 1.02±0.71 | |  | 0.150±0.152 | 0.143±0.154 |
| Q3 | | 51 | 0.87±0.66 | |  | Reference | Reference |
| Q4 | | 56 | 0.77±0.66 | |  | -0.108±0.154 | -0.108±0.156 |
| Q5 | | 55 | 1.06±1.01 | |  | 0.182±0.154 | 0.199±0.157 |
| C22:4n-6 (ADA) | | | | | 0.141±0.058^6^ |  |  |
| Q1 | | 54 | 0.74±0.61 | |  | -0.401±0.153^6^ | -0.383±0.157^6^ |
| Q2 | | 56 | 0.91±0.64 | |  | -0.229±0.152 | -0.203±0.154 |
| Q3 | | 54 | 1.14±0.89 | |  | Reference | Reference |
| Q4 | | 55 | 1.10±0.80 | |  | -0.046±0.153 | 0.010±0.157 |
| Q5 | | 55 | 1.04±0.97 | |  | -0.102±0.153 | -0.07±0.156 |
| C22:5n-6 (OA) | | |  | | 0.354±0.145^6^ |  |  |
| Q1 | | 55 | 1.22±1.08 | |  | 0.451±0.145^6^ | 0.490±0.149^6^ |
| Q2 | | 59 | 0.88±0.63 | |  | 0.115±0.142 | 0.134±0.144 |
| Q3 | | 57 | 0.73±0.46 | |  | Reference | Reference |
| Q4 | | 47 | 0.82±0.73 | |  | 0.071±0.153 | 0.063±0.158 |
| Q5 | | 56 | 1.29±0.87 | |  | 0.539±0.146^6^ | 0.574±0.150^6^ |
| Total n-6 PUFAs | | | | | -0.002 ±0.013 |  |  |
| Q1 | | 55 | 0.88±0.63 | |  | -0.287±0.153 | -0.322±0.156^6^ |
| Q2 | | 55 | 1.00±0.83 | |  | -0.162±0.153 | -0.202±0.157 |
| Q3 | | 54 | 1.16±0.86 | |  | Reference | Reference |
| Q4 | | 55 | 0.83±0.65 | |  | -0.337±0.153^6^ | -0.350±0.158^6^ |
| Q5 | | 55 | 1.06±0.98 | |  | -0.101±0.153 | -0.119±0.156 |
| C18:3n-3 (LNA) | | | | | 0.774±0.534 |  |  |
| Q1 | | 48 | 0.19±0.95 | |  | -0.469±0.174^6^ | 0.465±0.178^6^ |
| Q2 | | 81 | 0.97±0.66 | |  | 0.256±0.158 | 0.248±0.160 |
| Q3 | | 37 | 0.72±0.54 | |  | Reference | Reference |
| Q4 | | 38 | 0.81±0.77 | |  | 0.093±0.183 | 0.070±0.186 |
| Q5 | | 70 | 1.10±0.93 | |  | 0.382±0.161^6^ | 0.373±0.165^6^ |
| \| C18:4n-3 (SDA) \|  \| \| --- \| --- \| | | |  | | 0.001±0.195 |  |  |
| Q1 | | 58 | 1.13±0.89 | |  | 0.384±0.148^6^ | 0.399±0.150^6^ |
| Q2 | | 52 | 1.11±0.72 | |  | 0.365±0.152 | 0.383±0.156^6^ |
| Q3 | | 54 | 0.75±0.64 | |  | Reference | Reference |
| Q4 | | 60 | 0.75±0.64 | |  | 0.005±0.147 | 0.020±0.148 |
| Q5 | | 50 | 1.22±0.99 | |  | 0.473±0.154^6^ | 0.489±0.155^6^ |
| C20:3n-3 (EA) | | |  | | 0.054±0.032 |  |  |
| Q1 | | 55 | 0.70±0.82 | |  | -0.113±0.150 | -0.070±0.154 |
| Q2 | | 57 | 0.98±0.60 | |  | 0.173±0.149 | 0.183±0.151 |
| Q3 | | 52 | 0.81±0.69 | |  | Reference | Reference |
| Q4 | | 56 | 1.06±0.76 | |  | 0.246±0.149 | 0.265±0.151 |
| Q5 | | 54 | 1.38±0.95 | |  | 0.571±0.151^6^ | 0.584±0.152^6^ |
| C20:5n-3 (EPA) | | | | | 0.054±0.047 |  |  |
| Q1 | | 56 | 1.21±0.82 | |  | 0.250±0.148 | 0.243±0.152 |
| Q2 | | 54 | 0.97±0.71 | |  | 0.011±0.150 | 0.002±0.152 |
| Q3 | | 55 | 0.96±0.71 | |  | Reference | Reference |
| Q4 | | 54 | 0.61±0.54 | |  | -0.347±0.150^6^ | -0.361±0.152^6^ |
| Q5 | | 55 | 1.17±1.03 | |  | -0.207±0.149 | 0.210±0.151 |
| C22:5n-3 (DPA) | | | | | 0.375±0.197^6^ |  |  |
| Q1 | | 60 | 0.80±0.58 | |  | -0.117±0.132 | -0.110±0.134 |
| Q2 | | 50 | 0.61±0.47 | |  | -0.305±0.139^6^ | -0.311±0.139^6^ |
| Q3 | | 64 | 0.92±0.70 | |  | Reference | Reference |
| Q4 | | 51 | 1.01±0.96 | |  | 0.091±0.138 | 0.101±0.139 |
| Q5 | | 49 | 1.66±0.87 | |  | 0.741±0.139^6^ | 0.754±0.140^6^ |
| C22:6n-3 (DHA) | | | | | 0.112±0.039^6^ |  |  |
| Q1 | | 54 | 0.72±0.57 | |  | -0.330±0.147^6^ | -0.348±0.149^6^ |
| Q2 | | 54 | 0.69±0.47 | |  | -0.369±0.147^6^ | -0.369±0.150^6^ |
| Q3 | | 55 | 1.05±0.79 | |  | Reference | Reference |
| Q4 | | 56 | 1.37±0.88 | |  | 0.312±0.146^6^ | 0.310±0.148^6^ |
| Q5 | | 55 | 1.09±0.99 | |  | 0.035±0.147 | 0.039±0.149 |
| Total n-3 PUFAs | | | | | 6.146±1.767^6^ |  |  |
| Q1 | | 54 | 0.70±0.54 | |  | -0.464±0.151^6^ | -0.452±0.154^6^ |
| Q2 | | 55 | 0.89±0.70 | |  | -0.277±0.151^6^ | -0.264±0.154 |
| Q3 | | 55 | 1.16±0.83 | |  | Reference | Reference |
| Q4 | | 54 | 1.02±0.84 | |  | -0.140±0.151 | -0.118±0.154 |
| Q5 | | 56 | 1.15±0.84 | |  | -0.010±0.150 | 0.000±0.154 |
| Total n-6/n-3 PUFA radio | | | | | -0.163±0.043^6^ |  |  |
| Q1 | 55 | | 1.35±0.93 | |  | 0.385±0.149^6^ | 0.389±0.152^6^ |
| Q2 | 54 | | 0.95±0.84 | |  | -0.007±0.150 | -0.002±0.151 |
| Q3 | 55 | | 0.96±0.77 | |  | Reference | Reference |
| Q4 | 57 | | 0.97±0.78 | |  | 0.006±0.148 | 0.010±0.152 |
| Q5 | 53 | | 0.70±0.52 | |  | -0.265±0.151 | -0.242±0.153 |

^1^ All values are ‾X±SD.

^2^ All values are B±SE. B means β-coefficients from the regression model.

^3^ Continuous model with the SD score as fatty acid concentrations. B±SE is the unstandardized regression coefficient, representing changes in TL with 1-SD increase in fatty acid concentration.

^4^ Categorical model with quintiles as fatty acid concentrations. B±SE is the unstandardized regression coefficient, representing differences between TL in the specific quintile and that in the reference Q3 quintile.

^5^ Adjusted for gestational age at birth, infant sex, birth weight and length, and maternal age, maternal pre-pregnancy BMI, and gestational weight gain, and paternal age and BMI.

^6^ P <0.05.

**Table 6. Associations between fatty acids in maternal erythrocytes and telomere length in placenta (n=165).**

| Fatty acids | | n | TL^1^ | | | Univariate model^2^ | | | | Multivariate model^2,4,5^ | |
| --- | --- | --- | --- | --- | --- | --- | --- | --- | --- | --- | --- |
|  |  |  |  |  |  | Continuous^3^ | | Categorical^4^ | |  |  |
| C18:2n-6 (LA) | | |  | | | 0.008±0.011 | |  | |  | |
| Q1 | | 33 | 0.92±0.69 | | |  | | 0.085±0.145 | | 0.119±0.144 | |
| Q2 | | 41 | 0.97±0.56 | | |  | | 0.140±0.145 | | 0.130±0.145 | |
| Q3 | | 34 | 0.82±0.32 | | |  | | Reference | | Reference | |
| Q4 | | 38 | 1.15±0.75 | | |  | | 0.334±0.143^6^ | | 0.319±0.145^6^ | |
| Q5 | | 19 | 0.91±0.34 | | |  | | 0.095±0.144 | | 0.120±0.146 | |
| C18:3n-6 (GLA) | | | | | | -0.379±0.086^6^ | |  | |  | |
| Q1 | | 56 | 1.19±0.79 | | |  | | 0.155±0.118 | | 0.168±0.118 | |
| Q2 | | - | - | | |  | | - | | - | |
| Q3 | | 42 | 1.02±0.42 | | |  | | Reference | | Reference | |
| Q4 | | 31 | 0.84±0.37 | | |  | | -0.172±0.136 | | -0.188±0.135 | |
| Q5 | | 36 | 0.67±0.35 | | |  | | -0.361±0.136^6^ | | -0.371±0.134^6^ | |
| C20:3n-6 (DGLA) | | | | | | -0.266±0.130^6^ | |  | |  | |
| Q1 | | 34 | 1.09±0.71 | | |  | | 0.115±0.145 | | 0.051±0.146 | |
| Q2 | | 37 | 0.98±0.63 | | |  | | 0.008±0.147 | | -0.069±0.148 | |
| Q3 | | 34 | 0.97±0.66 | | |  | | Reference | | Reference | |
| Q4 | | 36 | 0.88±0.45 | | |  | | -0.101±0.145 | | -0.201±0.148 | |
| Q5 | | 24 | 0.88±0.40 | | |  | | -0.079±0.145 | | -0.123±0.145 | |
| C20:4n-6 (AA) | | | | | | -0.041±0.024 | |  | |  | |
| Q1 | | 29 | 1.14±0.75 | | |  | | 0.148±0.145 | | 0.156±0.145 | |
| Q2 | | 27 | 1.01±0.40 | | |  | | 0.024±0.144 | | 0.041±0.142 | |
| Q3 | | 36 | 0.99±0.64 | | |  | | Reference | | Reference | |
| Q4 | | 33 | 0.81±0.49 | | |  | | -0.165±0.144 | | -0.177±0.143 | |
| Q5 | | 40 | 0.92±0.57 | | |  | | -0.067±0.144 | | -0.075±0.144 | |
| C22:2n-6 (DDA) | | | | |  | -0.104±0.103 | |  | |  | |
| Q1 | | 36 | 1.12±0.73 | | |  | | 0.146±0.145 | | 0.146±0.147 | |
| Q2 | | 35 | 0.94±0.53 | | |  | | -0.047±0.144 | | -0.016±0.144 | |
| Q3 | | 28 | 0.99±0.51 | | |  | | Reference | | Reference | |
| Q4 | | 37 | 0.80±0.52 | | |  | | -0.191±0.143 | | -0.190±0.145 | |
| Q5 | | 29 | 0.99±0.59 | | |  | | -0.001±0.145 | | -0.018±0.145 | |
| C22:4n-6 (ADA) | | | | | | -0.150±0.076^6^ | |  | |  | |
| Q1 | | 27 | 1.22±0.75 | | |  | | 0.269±0.143^6^ | | 0.309±0.142^6^ | |
| Q2 | | 27 | 0.96±0.40 | | |  | | 0.032±0.142 | | 0.037±0.140 | |
| Q3 | | 37 | 0.94±0.66 | | |  | | Reference | | Reference | |
| Q4 | | 39 | 0.97±0.64 | | |  | | 0.041±0.142 | | 0.045±0.142 | |
| Q5 | | 35 | 0.80±0.35 | | |  | | -0.131±0.141 | | -0.123±0.140 | |
| C22:5n-6 (OA) | | | | | | -0.584±0.187^6^ | |  | |  | |
| Q1 | | 23 | 1.40±1.12 | | |  | | 0.350±0.154^6^ | | 0.309±0.161 | |
| Q2 | | 40 | 0.94±0.44 | | |  | | -0.113±0.134 | | -0.104±0.135 | |
| Q3 | | 31 | 1.05±0.37 | | |  | | Reference | | Reference | |
| Q4 | | 38 | 0.84±0.46 | | |  | | -0.205±0.136 | | -0.235±0.138 | |
| Q5 | | 33 | 0.76±0.32 | | |  | | -0.286±0.140^6^ | | -0.261±0.143 | |
| Total n-6 PUFAs | | | | | | | -0.018±0.010 |  |  | |  |
| Q1 | 34 | | | 1.09±0.56 | | |  | 0.075±0.144 | 0.103±0.145 | |  |
| Q2 | 37 | | | 0.95±0.76 | | |  | -0.061±0.146 | -0.021±0.146 | |  |
| Q3 | 41 | | | 1.00±0.66 | | |  | Reference | Reference | |  |
| Q4 | 34 | | | 0.85±0.40 | | |  | -0.168±0.145 | -0.159±0.147 | |  |
| Q5 | 19 | | | 0.92±0.34 | | |  | -0.084±0.146 | -0.056±0.149 | |  |
| C18:3n-3 (LNA) | | | | | | -0.477±0.166^6^ | |  | |  | |
| Q1 | | 30 | 0.88±0.61 | | |  | | -0.221±0.135 | | -0.209±0.136 | |
| Q2 | | 39 | 1.17±0.55 | | |  | | 0.073±0.126 | | 0.061±0.126 | |
| Q3 | | 42 | 1.10±0.73 | | |  | | Reference | | Reference | |
| Q4 | | 31 | 0.84±0.33 | | |  | | -0.257±0.134 | | -0.272±0.136^6^ | |
| Q5 | | 23 | 0.65±0.42 | | |  | | -0.449±0.147^6^ | | -0.469±0.147^6^ | |
| C18:4n-3 (SDA) | | | | | | -0.278±0.166 | |  | |  | |
| Q1 | | 39 | 1.06±0.67 | | |  | | 0.252±0.146 | | 0.237±0.147 | |
| Q2 | | 34 | 0.98±0.62 | | |  | | 0.164±0.146 | | 0.150±0.146 | |
| Q3 | | 25 | 0.76±0.53 | | |  | | Reference | | Reference | |
| Q4 | | 34 | 1.07±0.63 | | |  | | 0.263±0.148 | | 0.209±0.149 | |
| Q5 | | 33 | 0.89±0.41 | | |  | | 0.081±0.145 | | 0.061±0.147 | |
| C20:3n-3 (EA) | | |  | | | -0.081±0.040^6^ | |  | |  | |
| Q1 | | 38 | 1.03±0.59 | | |  | | -0.108±0.144 | | -0.095±0.145 | |
| Q2 | | 42 | 1.01±0.62 | | |  | | -0.130±0.141 | | -0.110±0.142 | |
| Q3 | | 34 | 1.10±0.65 | | |  | | Reference | | Reference | |
| Q4 | | 31 | 0.87±0.54 | | |  | | -0.273±0.140^6^ | | -0.275±0.141^6^ | |
| Q5 | | 20 | 0.66±0.36 | | |  | | -0.412±0.142^6^ | | -0.391±0.144^6^ | |
| C20:5n-3 (EPA) | | | | | | 0.006±0.100 | |  | |  | |
| Q1 | | 36 | 1.03±0.66 | | |  | | 0.023±0.146 | | 0.015±0.147 | |
| Q2 | | 38 | 0.91±0.44 | | |  | | -0.102±0.142 | | -0.118±0.144 | |
| Q3 | | 22 | 1.02±0.71 | | |  | | Reference | | Reference | |
| Q4 | | 32 | 0.75±0.41 | | |  | | -0.261±0.144 | | -0.289±0.144^6^ | |
| Q5 | | 37 | 1.12±0.67 | | |  | | 0.118±0.143 | | 0.080±0.144 | |
| C22:5n-3 (DPA) | | | | | | -0.304±0.103^6^ | |  | |  | |
| Q1 | | 21 | 1.05±0.54 | | |  | | 0.096±0.156 | | 0.174±0.158 | |
| Q2 | | 38 | 0.26±0.84 | | |  | | 0.307±0.133^6^ | | 0.333±0.133^6^ | |
| Q3 | | 34 | 0.95±0.40 | | |  | | Reference | | Reference | |
| Q4 | | 36 | 0.88±0.52 | | |  | | -0.073±0.135 | | -0.057±0.134 | |
| Q5 | | 36 | 0.71±0.33 | | |  | | -0.242±0.135 | | -0.227±0.093 | |
| C22:6n-3 (DHA) | | | | | | -0.161±0.049^6^ | |  | |  | |
| Q1 | | 39 | 1.15±0.74 | | |  | | 0.079±0.140 | | 0.048±0.139 | |
| Q2 | | 35 | 1.04±0.50 | | |  | | -0.061±0.139 | | -0.045±0.139 | |
| Q3 | | 28 | 1.10±0.68 | | |  | | Reference | | Reference | |
| Q4 | | 35 | 0.81±0.40 | | |  | | -0.295±0.140^6^ | | -0.304±0.139^6^ | |
| Q5 | | 28 | 0.68±0.40 | | |  | | -0.392±0.139^6^ | | -0.413±0.139^6^ | |
| Total n-3 PUFAs | | | | | | | -8.437±2.236^6^ |  |  | |  |
| Q1 | 46 | | | 1.18±0.67 | | |  | 0.260±0.140^6^ | 0.238±0.139 | |  |
| Q2 | 30 | | | 1.05±0.73 | | |  | 0.082±0.141 | 0.034±0.142 | |  |
| Q3 | 32 | | | 0.92±0.36 | | |  | Reference | Reference | |  |
| Q4 | 28 | | | 0.84±0.56 | | |  | -0.111±0.141 | -0.135±0.141 | |  |
| Q5 | 29 | | | 0.72±0.37 | | |  | -0.226±0.141 | -0.259±0.141 | |  |
| Total n-6/n-3PUFA radio | | | | | | 0.094±0.036^6^ | |  | |  | |
| Q1 | | 24 | 0.72±0.42 | | |  | | -0.153±0.141 | | -0.122±0.141 | |
| Q2 | | 37 | 0.88±0.48 | | |  | | -0.037±0.142 | | -0.052±0.142 | |
| Q3 | | 37 | 0.90±0.38 | | |  | | Reference | | Reference | |
| Q4 | | 34 | 1.20±0.80 | | |  | | 0.289±0.140^6^ | | 0.276±0.140^6^ | |
| Q5 | | 33 | 1.07±0.66 | | |  | | 0.163±0.141 | | 0.205±0.141 | |

^1^ All values are ‾X±SD.

^2^ All values are B±SE. B means β-coefficients from the regression model.

^3^ Continuous model with the SD score as fatty acid concentrations. B±SE is the unstandardized regression coefficient, representing changes in TL with 1-SD increase in fatty acid concentration

^4^ Categorical model with quintiles as measure of fatty acids concentration. B±SE is the unstandardized regression coefficient, representing differences between TL in the specific quintile and that in the reference Q3 quintile.

^5^ Adjusted for gestational age at birth, infant sex, birth weight and length, and maternal age, pre-pregnancy BMI and gestational weight gain, and paternal age and BMI.

^6^ P <0.05.

**Table 7 Associations between methylation fractions of CpG sites in the TERT promoter and TL in the cord blood (n=177) and the placenta (n=165).**

| CpG sites | Umbilical cord blood^1^ | | Placenta^1^ | |
| --- | --- | --- | --- | --- |
|  | Univariate model^2^ | Multivariate model^3^ | Univariate model^2^ | Multivariate model^3^ |
| 1 | 0.007±0.008 | 0.007±0.008 | -0.003±0.004 | -0.002±0.004 |
| 2 | 0.020±0.008^4^ | 0.020±0.008^4^ | -0.007±0.004 | -0.007±0.004 |
| 3 | 0.027±0.007^4^ | 0.027±0.007^4^ | -0.004±0.004 | -0.004±0.004 |
| 4 | 0.006±0.007 | 0.005±0.007 | -0.006±0.004 | -0.005±0.004 |
| 5 | 0.016±0.007^4^ | 0.015±0.007^4^ | -0.005±0.004 | -0.004±0.004 |
| 6 | 0.013±0.007 | 0.011±0.007 | -0.007±0.005 | -0.005±0.004 |
| 7 | 0.011±0.007 | 0.010±0.008 | -0.004±0.004 | -0.004±0.004 |
| 8 | 0.008±0.006 | 0.007±0.006 | -0.001±0.004 | -0.001±0.003 |
| 9 | -0.001±0.006 | -0.002±0.006 | -0.001±0.004 | -0.001±0.004 |
| 10 | 0.003±0.006 | 0.002±0.006 | -0.002±0.004 | -0.002±0.003 |
| 11 | 0.007±0.006 | 0.006±0.006 | -0.001±0.004 | -0.002±0.003 |
| 12 | -0.004±0.007 | -0.007±0.007 | 0.000±0.004 | 0.000±0.003 |
| 13 | -0.004±0.007 | -0.004±0.007 | 0.005±0.004 | 0.004±0.004 |
| 14 | 0.002±0.007 | 0.001±0.007 | 0.008±0.004^4^ | 0.007±0.004^4^ |
| 15 | 0.010±0.006 | 0.009±0.007 | 0.001±0.004 | 0.001±0.003 |
| 16 | 0.012±0.006^4^ | 0.012±0.006^4^ | 0.004±0.003 | 0.005±0.003 |
| 17 | 0.011±0.006 | 0.011±0.006 | 0.002±0.004 | 0.002±0.003 |
| 18 | 0.009±0.005 | 0.010±0.006 | 0.000±0.004 | 0.001±0.003 |
| 19 | 0.003±0.006 | 0.002±0.006 | 0.001±0.004 | 0.002±0.003 |
| 20 | 0.004±0.005 | 0.004±0.005 | -0.002±0.003 | -0.001±0.003 |
| 21 | 0.007±0.005 | 0.007±0.005 | 0.001±0.004 | 0.001±0.003 |
| 22 | 0.006±0.006 | 0.007±0.006 | 0.002±0.003 | 0.003±0.003 |
| 23 | 0.008±0.006 | 0.008±0.006 | 0.001±0.006 | 0.001±0.006 |
| 24 | 0.018±0.006^4^ | 0.017±0.006^4^ | -0.005±0.006 | -0.007±0.006 |
| 25 | 0.007±0.008 | 0.007±0.008 | -0.015±0.008^4^ | -0.008±0.005 |
| Average | 0.018±0.010^4^ | 0.018±0.010^4^ | -0.001±0.005 | -0.001±0.005 |

^1^ All values are B±SE. B means β-coefficients from the regression model.

^2^ In the univariate model, B±SE is the unstandardized regression coefficient, representing changes in TL in the cord blood and the placenta with 1-SD increase in methylation fractions of CpG sites of the TERT promoter.

^3^ Adjusted for gestational age at birth, infant sex, birth weight and length, and maternal age, pre-pregnancy BMI and gestational weight gain, and paternal age and BMI.

^4^ P <0.05.

**Table 8 Associations between selected fatty acids and averaged methylation fractions of the TERT promoter in the cord blood (n=177) and the placenta (n=165).**

| Fatty acids | Umbilical cord blood^1^ | | | Placenta^1^ | | |
| --- | --- | --- | --- | --- | --- | --- |
|  | Univariate  model^2^ | | Multivariate model^3^ | | Univariate  model^2^ | Multivariate  model^3^ |
| C18:2n-6 (LA) | 0.087±0.105 | | 0.086±0.106 | | -0.209±0.264 | -0.260±0.269 |
| C18:3n-6 (GLA) | 0.004±0.896 | | 0.057±0.911 | | 3.156±1.425^4^ | 3.479±1.429^4^ |
| C20:3n-6 (DGLA) | 2.609±1.116^4^ | | 2.602±1.186^4^ | | 2.393±2.144 | 1.806 ±2.176 |
| C20:4n-6 (AA) | 0.115±0.229 | | 0.145±0.231 | | 0.509±0.353 | 0.364±0.361 |
| C22:2n-6 (DDA) | 2.097±0.967^4^ | | 2.719±1.066^4^ | | -3.001±1.580 | -3.736±1.602 |
| C22:4n-6 (ADA) | -0.008±0.732 | | 0.177±0.744 | | 2.342±1.260 | 1.792±1.326 |
| C22:5n-6 (OA) | 2.287±1.694 | | 2.070±1.707 | | 4.298±2.965 | 3.936±2.998 |
| Total n-6 PUFAs | 0.140±0.095 | | 0.156±0.098 | | 0.120±0.161 | 0.052±0.193 |
| C18:3n-3 (LNA) | 0.536±1.386 | | 0.243±1.418 | | -0.462±2.367 | 0.283±2.406 |
| C18:4n-3 (SDA) | 5.456±1.908^4^ | | 5.856±1.930^4^ | | 0.467±2.486 | 0.155±2.519 |
| C20:3n-3  (EA) | 0.223±0.429 | | 0.171±0.444 | | 0.558±1.109 | 0.245±1.141 |
| C20:5n-3 (EPA) | 1.741±0.932^4^ | | 2.039±0.945^4^ | | -0.676±1.449 | -1.076±1.463 |
| C22:5n-3 (DPA) | 0.287±0.126 | | 0.514±1.138 | | 0.558±1.676 | 0.368±1.699 |
| C22:6n-3 (DHA) | 1.214±0.506^4^ | | 1.370±0.511^4^ | | 0.485±0.788 | 0.441±0.792 |
| Total n-3 PUFAs | 49.367±21.211^4^ | | 56.745±22.824^4^ | | 20.910±36.325 | 15.107±38.797 |
| Total n-6/n-3 PUFA radio | | -0.533±0.335 | -0.598±0.341 | | 0.293±0.569 | 0.293±0.606 |

^1^ All values are B±SE. B means β-coefficients from the regression model.

^2^ In the univariate model, B±SE is the unstandardized regression coefficient, representing the changes in averaged DNA methylation fractions of TERT promoter in the cord blood and placenta with a 1-SD increase in maternal fatty acids.

^3^ Adjusted for gestational age at birth, infant sex, birth weight and length, and maternal age, pre-pregnancy BMI and gestational weight gain, and paternal age and BMI.

^4^ P <0.05.
